# Integrating a tailored recurrent neural network with Bayesian experimental design to optimize microbial community functions

**DOI:** 10.1371/journal.pcbi.1011436

**Published:** 2023-09-29

**Authors:** Jaron C. Thompson, Victor M. Zavala, Ophelia S. Venturelli

**Affiliations:** 1 Department of Chemical and Biological Engineering, University of Wisconsin-Madison, Madison, Wisconsin, United States of America; 2 Department of Biochemistry, University of Wisconsin-Madison, Madison, Wisconsin, United States of America; 3 Department of Bacteriology, University of Wisconsin-Madison, Madison, Wisconsin, United States of America; CPERI, GREECE

## Abstract

Microbiomes interact dynamically with their environment to perform exploitable functions such as production of valuable metabolites and degradation of toxic metabolites for a wide range of applications in human health, agriculture, and environmental cleanup. Developing computational models to predict the key bacterial species and environmental factors to build and optimize such functions are crucial to accelerate microbial community engineering. However, there is an unknown web of interactions that determine the highly complex and dynamic behavior of these systems, which precludes the development of models based on known mechanisms. By contrast, entirely data-driven machine learning models can produce physically unrealistic predictions and often require significant amounts of experimental data to learn system behavior. We develop a physically-constrained recurrent neural network that preserves model flexibility but is constrained to produce physically consistent predictions and show that it can outperform existing machine learning methods in the prediction of certain experimentally measured species abundance and metabolite concentrations. Further, we present a closed-loop, Bayesian experimental design algorithm to guide data collection by selecting experimental conditions that simultaneously maximize information gain and target microbial community functions. Using a bioreactor case study, we demonstrate how the proposed framework can be used to efficiently navigate a large design space to identify optimal operating conditions. The proposed methodology offers a flexible machine learning approach specifically tailored to optimize microbiome target functions through the sequential design of informative experiments that seek to explore and exploit community functions.

## Author summary

The functions performed by microbiomes hold tremendous promise to address grand challenges facing society ranging from improving human health to promoting plant growth. To design their properties, flexible computational models that can predict the temporally changing behaviors of microbiomes in response to key environmental parameters are needed. When considering a bottom-up design of microbiomes, the number of possible communities grows exponentially with the number of organisms and environmental factors, which makes it challenging to navigate the microbiome function landscape. To overcome these challenges, we present a physically-constrained machine learning model for microbiomes and a closed-loop, Bayesian experimental design framework to efficiently navigate the space of possible communities and environmental factors.

## 1 Introduction

Microbial communities have the potential to perform a variety of functions, including the ability to convert carbon-rich waste products into valuable compounds [1, 2], perform biological nitrogen fixation to improve agricultural yields [3], detoxify waste from the environment [4], and modulate vertebrate host phenotypes [5]. However, designing microbial communities from the bottom-up to perform desired functions is a major challenge due to unknown mechanisms of interaction and limited ability to observe and quantify all aspects of such systems (e.g. metabolites utilized and produced by constituent community members). Further, the design space of species and environmental factors for optimizing a microbiome target function is large and difficult to systematically navigate. Developing models that predict the temporal behaviors of communities from data and identify environmental conditions and combinations of species predicted to have optimized functions has emerged as a promising avenue to direct microbiome engineering [6].

Because microbiomes have large design spaces, high-throughput experiments coupled to computational modeling can be powerful for understanding and engineering microbial communities from the bottom-up [7–9]. Mathematical models that predict system behavior have become essential tools to understand complex biological processes [10], and recent studies have successfully applied a model guided approach to understand and optimize microbial community functions [5, 9]. Developing models of the microbiome from first-principles is difficult due to unknown interactions as well as a limited understanding of the mechanisms that underlie these interactions [11]. Machine learning methods that can learn how microbial species interact in different environments from experimental data are thus compelling approaches to address this limitation. Neural networks are flexible machine learning models that can predict complex behavior for a broad class of systems [12]. Recurrent neural networks (RNNs), in particular, are powerful neural network architectures that can exploit multivariate time series data to learn dynamic behaviors [13]. For example, Baranwal et al. [14] showed that RNNs could model microbial community dynamics with greater accuracy than standard ecological models that are confined by a strict set of assumptions, such as the generalized Lotka-Volterra (gLV) model. In addition to improved prediction performance of species growth dynamics, the model was able to accurately forecast the production of health relevant metabolites given an initial profile of species abundances. In addition, an RNN model trained on time series measurements of human gut microbiome composition data tailored for classification of food allergy achieved the best prediction accuracy compared to other machine learning methods [15].

While highly flexible, key limitations of applying machine learning models such as RNNs to physical systems include unrealistic predictions (e.g. negative species abundances) and significant amounts of experimental data for training. Machine learning models are capable of making unrealistic predictions when the training data set (i.e. data used to build the model) is insufficient to constrain the model to match system behavior. Physically constrained machine learning models are especially promising for modeling biological systems [16] because these constraints can potentially improve a model’s ability to extrapolate beyond the regime explored in the training set despite limited or noisy data [17, 18]. In the computational biology field, for instance, neural networks have been used in concert with mechanistic ordinary differential equation (ODE) models for system identification of hidden dynamics of experimentally unobserved variables in biological systems [19]. In addition to incorporating physical constraints, experimental design strategies that optimize the information content of experimental data can reduce the amount of data needed to train a predictive model.

The collection of data used to inform machine learning models requires taking measurements of system properties, which is often time-consuming and expensive. Consequently, the selection of an informative set of experiments is crucial for developing models that capture system properties, while minimizing time and resources spent on performing experiments [20]. To achieve this goal, determining an optimal set of experiments that minimizes either model prediction uncertainty or uncertainty in parameter estimates has been widely used to optimize the information content of experiments for studying biological systems [21–24]. Bayesian experimental design naturally integrates previously observed data to inform the selection of new experimental conditions. This enables a sequential strategy that uses all previously collected data to inform future iterations of model fitting, experimental design, and data acquisition. These approaches use acquisition functions that aim to quantify information content and predict system performance under potential sets of experiments. A widely used acquisition function is called the expected information gain (EIG), which quantifies how well an experimental design is expected to constrain estimates of model parameters [24–26]. While the EIG provides a principled acquisition function to design new experimental conditions, it is typically intractable to compute analytically for nonlinear models and can be computationally expensive to evaluate when approximate approaches such as Monte Carlo sampling [27] are used.

While most applications of Bayesian experimental design have focused on conducting experiments to refine a model, experimental design strategies have rarely been used in the field of systems biology for the purpose of seeking conditions that optimize properties of the system (e.g. production of a valuable compound or pathogen inhibition) for target applications. However, Bayesian experimental design can be tailored to provide a powerful goal-oriented framework that can leverage a flexible class of models to propose experimental conditions that have the dual objective of mitigating model uncertainty and optimizing system performance [25, 28]. For example, Bayesian optimization is a closed-loop experimental design technique whose purpose is to efficiently optimize system properties and has been used in many fields ranging from synthetic biology [29] to aerospace engineering [30]. Bayesian optimization typically uses a non-parametric Gaussian process model to predict system performance directly from experimental data. While Gaussian process models provide a natural and computationally-tractable approach to construct acquisition functions [31], they cannot easily model the dynamic behavior of multivariate systems [32]. Another widely used goal-oriented experimental design strategy is called response surface methodology, which proposes experiments to build a performance function that is optimized to find the best operating conditions. However, this approach is typically limited to linear models [33] and experimental designs are based on rigidly defined structures [34].

We address gaps in model-guided experimental design of microbial communities by developing and applying a physically-constrained RNN architecture tailored to predict microbial community dynamics and target functions (e.g. production of specific metabolites) in response to environmental inputs. The proposed model outperforms other representative machine learning methods in the prediction of species abundances and metabolite concentrations using experimental data composed of unique human gut communities (> 10 species). Equipped with this model, we present a closed-loop, Bayesian experimental design framework to optimize microbial community functions that leverages an information theoretic approach to select a set of experimental conditions that collectively exploit system functions and fill knowledge gaps in the model. We demonstrate the capability of the overall framework to minimize the number of experiments necessary to identify optimal operating conditions that maximize production of a desired metabolite using a mechanistic multi-species microbial community model. To our knowledge, our framework is the first to integrate sequential Bayesian experimental design to optimize dynamic system behaviors using a RNN based model. Furthermore, we present a novel RNN architecture that is specifically tailored to capture microbiome behaviors and process microbiome data.

## 2 Results

### 2.1 Design of microbial communities using a physically constrained recurrent neural network

**Fig 1:**
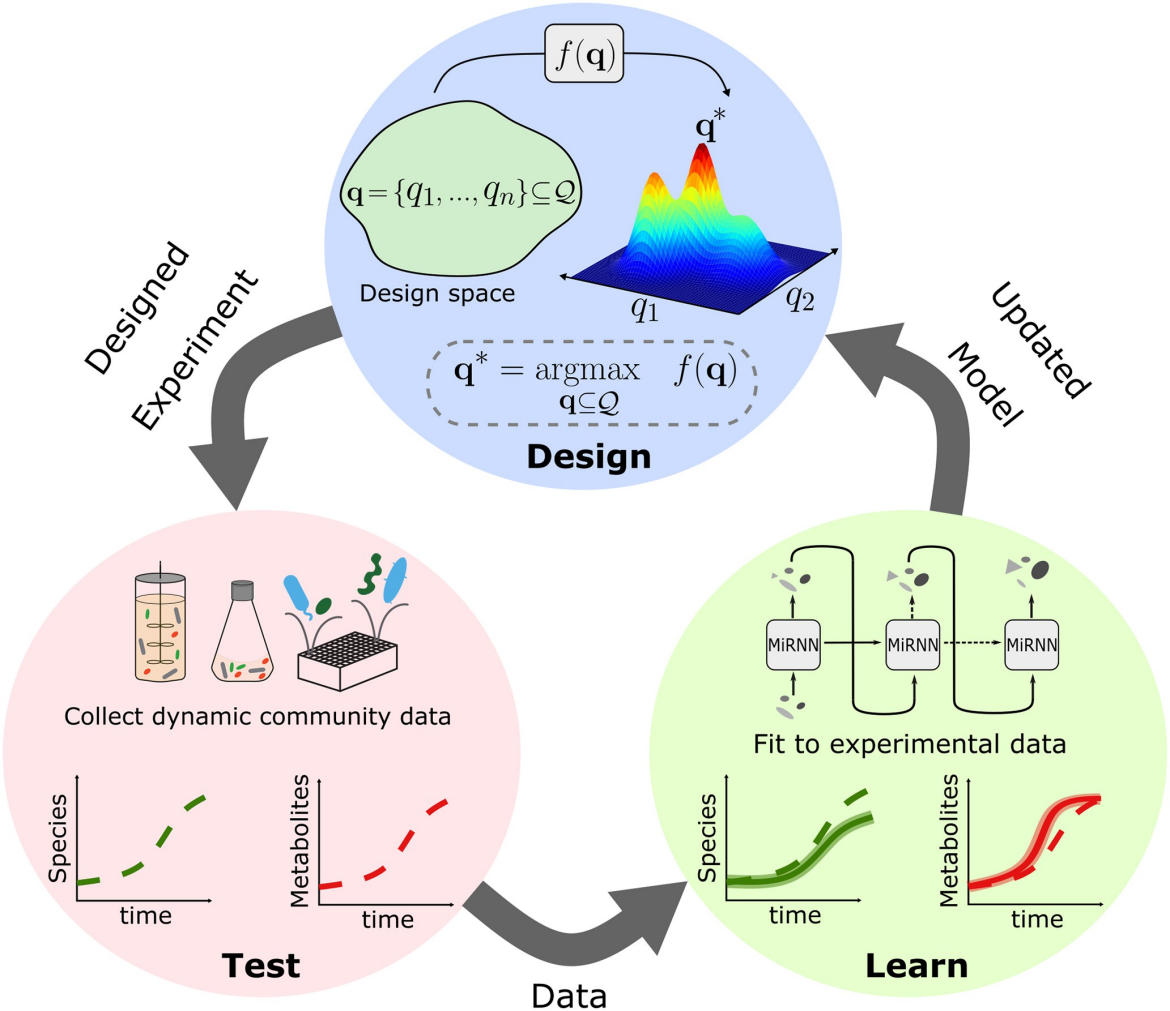
The Microbiome Recurrent Neural Network (MiRNN) learns system dynamics and proposes new designs. (**Design**) An experimental design space, denoted as Q, is a set of individual experimental conditions, **q**, where a particular condition could, for example, be a set of species in a community or the initial concentrations of resources. MiRNN predictions of outcomes for a set of experimental conditions, **q**, are evaluated by an acquisition function, *f*, which balances the expected information gain (EIG) of an experimental design and its expected profit to evaluate the optimality of experimental designs. (**Test**) The optimal experimental design, q*, defines a set of experimental conditions to be observed experimentally. Measurements of these conditions are collected in the test phase. (**Learn**) Data collected in the test phase, and all previously collected data, are used to fit an updated MiRNN model. Once fit to the newly acquired data, the updated MiRNN model can be used again in the design phase to complete the design, test, learn cycle.

Machine learning models can generate physically unrealistic predictions for physical systems. To address this limitation, we present the Microbiome Recurrent Neural Network (MiRNN), a modified RNN architecture that eliminates the possibility of predicting physically unrealistic species abundances and metabolite concentrations (S1 Fig). We leverage a Bayesian inference method for parameter estimation, hyper-parameter optimization, and quantification of prediction uncertainty. A model-guided approach is used to identify a set of experimental conditions that collectively maximize information content of different experimental designs and design objectives. Our closed-loop framework allows for the selection of an optimal set of experimental conditions that are collectively informative, as opposed to the selection of a single experimental condition, enabling design of high-throughput experiments. The proposed methodology is illustrated in Fig 1. In the design phase, the MiRNN is combined with an acquisition function, *f*, to rank experimental designs based on predicted outcomes and the expected information gain (EIG) from a space of all possible experimental conditions, denoted as Q. The acquisition function is composed of two parts, one that quantifies the expected profit (i.e. exploitation) of an experimental design and one that quantifies the information content (i.e. exploration) of an experimental design. The highest ranked design, $q^{*}\subseteq Q$, is tested to generate experimental data in the test phase. The resulting data is used to update the MiRNN model in the learn phase. The updated model is used to design the next experiment, completing the design, test, learn (DTL) cycle. DTL cycles can be repeated until convergence or until a desired objective is achieved.

### 2.2 Comparison of the Microbiome Recurrent Neural Network (MiRNN) model to an unconstrained recurrent neural network

Microbiome data of both synthetic and natural microbiomes are often sparse, meaning that most taxa are absent from community samples depending on the phylogenetic resolution [35]. The constraint embedded in the MiRNN is designed to account for this sparsity when making predictions of microbial community dynamics. Mirroring recurrent neural network based models [14], the MiRNN takes as input an initial condition of known species abundances and predicts future time points autoregressively. This involves making predictions at each time step, which in turn are model inputs to predict the next time step. Therefore, it is important that the model does not erroneously predict the emergence of an absent species at any step, since the error could propagate into predictions of subsequent time points. To prevent physically unrealistic predictions, the MiRNN includes a constraint that forces zero abundance species to remain zero at later time points and a rectified linear unit (ReLU) output activation that ensures species and metabolite predictions are non-negative. In contrast, an entirely unconstrained model could predict negative species abundances and metabolite concentrations or spontaneous appearance of species that were not initially present (S2 Fig).

**Fig 2:**
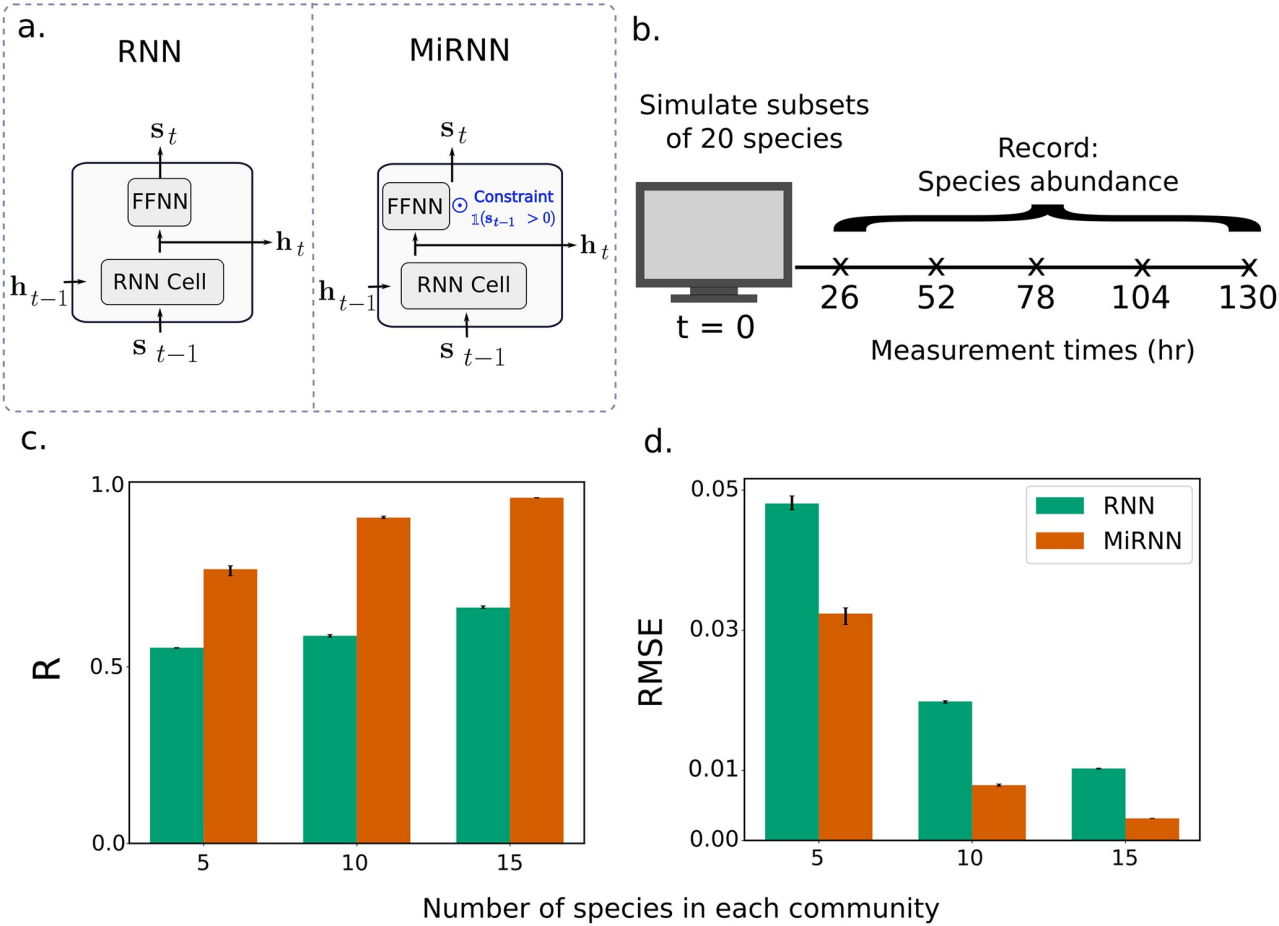
The predictive capability of the MiRNN outperforms an unconstrained RNN model using simulated data over a range of sparsity levels. (a.) A comparison of the MiRNN architecture to a standard RNN, where the constraint highlighted in blue prevents the model from predicting the spontaneous emergence of a species. (b.) Schematic of simulated data generation, indicating that a ground truth computational bioreactor model is used to simulate species abundances over a time span of 130 hours, with measurements of species abundances taken at 26 hour intervals. (c.) Comparison of RNN (green) and MiRNN (orange) performance in species predictions according to the average Pearson correlation coefficient (R) over all species between predictions and measured values. The height of the bars and error bars correspond to the median and interquartile range in prediction performance after running 10-fold cross-validation over 5 trials, where samples were randomly shuffled in each trial. (d.) Same as in panel (c.), except that RMSE instead of Pearson correlation is shown.

To demonstrate the utility of the constraint, we compared the MiRNN to an unconstrained RNN, which did not include the constraint to prevent the emergence of absent species (Fig 2A). All other aspects, including the ReLU output activation and the training algorithm (Algorithm 1), were exactly the same between the two models. We constructed a ground truth bioreactor model (Methods) of 20 species competing for 10 resources and considered three different simulated data sets: (1) 50 randomly selected community combinations of 5 of the 20 possible species (most sparse), (2) 50 combinations of 10 of the 20 possible species (intermediate sparsity), and (3) 50 combinations of 15 of the 20 possible species (least sparse). For each community combination, the ground truth model was simulated over a time span of 130 hours with observations of species abundances taken every 26 hours for a total of 5 time point measurements (Fig 2B). To investigate differences in prediction performance of the MiRNN and RNN models, we performed 10-fold cross-validation by randomly partitioning the data into 10 unique sets of samples, training on 9 subsets, testing on the remaining subset, and then repeating for each combination of training and testing data so that all samples were subject to held-out testing. Because the partitioning of the data subsets is random, we repeated cross-validation over 5 trials to evaluate the variation in prediction performance. We note that evaluations of prediction performance only consider predictions of species that were initially present, so that predictions of absent species do not bias benchmarks of model prediction performance in favor of the MiRNN. The MiRNN outperformed the RNN in terms of the average Pearson correlation and RMSE of predicted species abundances (Fig 2C and 2D) in all three data sets. Both models became increasingly more accurate as the sparsity reduced, highlighting the importance of models that account for sparsity in microbiome data. These results demonstrate that constraining absent species to remain zero at future time steps improved the MiRNN’s prediction accuracy of species that were initially present, making the MiRNN specifically tailored to process sparse microbiome data.

**Fig 3:**
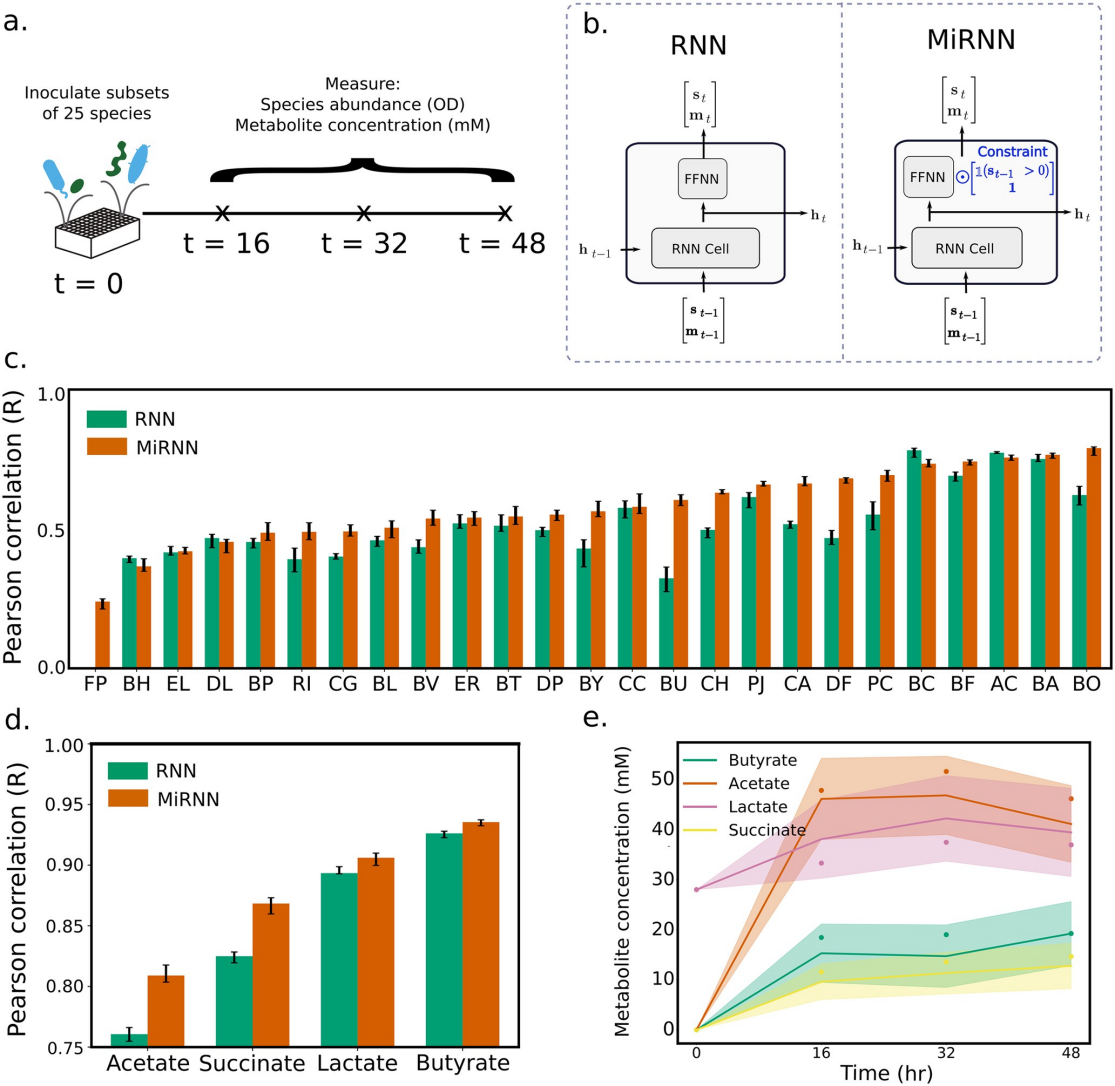
The predictive capability of the MiRNN outperforms an unconstrained RNN model using experimental data. (a.) Schematic of experiment in which 95 unique microbial consortia were selected from a set of 25 health-relevant human gut bacteria. After inoculation, species abundances and metabolite concentrations were measured at 16 hour intervals over a course of 48 hours. (b.) A comparison of the MiRNN architecture to a standard RNN, where the constraint highlighted in blue prevents the model from predicting the spontaneous emergence of a species. (c.) Comparison RNN and MiRNN performance in species predictions according to the Pearson correlation coefficient between predictions and measured values. The height of the bars and error bars correspond to the median and interquartile range in prediction performance after running 20-fold cross-validation over 10 trials, where samples were randomly shuffled in each trial. (d.) Same as in panel (c.), except that metabolite prediction performance is shown. (e.) Representative temporal changes in MiRNN predicted metabolite concentrations, where measured values are shown as dots, the mean predicted value is shown as a line, and the uncertainty region shows ± 1 standard deviation.

Microbiomes produce and degrade a myriad of metabolites, which mediate interactions with constituent community members and can be exploited to our benefit. To test the ability of the MiRNN to predict species abundance and metabolite concentration over time, we evaluated the model’s prediction performance on experimental data in which the absolute abundances of 25 diverse and prevalent human gut species and the concentrations of four major health-relevant metabolites (acetate, butyrate, lactate and succinate) were measured over time [9, 14] (Fig 3A). In particular, butyrate produced by gut microbiota has beneficial effects on human health and disease, including promoting homeostasis in the colon [36, 37] and protecting against metabolic disorders [38]. The ability to predict metabolite concentrations such as butyrate as a function of the initial abundance could inform the design of next-generation defined bacterial therapeutics. To test our model’s predictive capability, we used an experimental data set consisting of 95 unique subsets of the 25 member community that were inoculated in equal species proportions *in vitro*. Species abundances and metabolite concentrations were measured every 16 hours for a total of 48 hours to characterize community assembly and metabolite dynamics.

To investigate differences in prediction performance of the MiRNN and RNN models (Fig 3B), we performed 10 trials of 20-fold cross-validation by randomly partitioning the data into 20 unique sets of samples, training on 19 subsets and testing on the remaining subset. This was repeated for each combination of training and testing data so that all samples were subject to held-out testing. On held-out data, MiRNN predictions of species abundances that were initially present displayed a higher median Pearson correlation than the unconstrained RNN for 21 of the 25 species (Fig 3C), indicating that the incorporation of a physical constraint significantly improved the model’s ability to predict species abundance (sign test, *p* < 1 × 10^−3^, *n* = 25). Although the constraint does not directly impact predictions of metabolite concentrations, the MiRNN outperformed the RNN in predictions of acetate and succinate. The MiRNN and RNN displayed similar prediction performance of lactate and butyrate, which displayed the highest prediction performance of the four metabolites (Fig 3D). A representative trajectory shows the predicted distribution (mean ± 1 standard deviation) of each metabolite compared to measured values (Fig 3E).

We also compared the prediction performance of the MiRNN to a consumer resource (CR) model [39] and to the Long-Short-Term-Memory (LSTM) model developed by Baranwal et al. [14]. The LSTM model was previously shown to accurately predict community dynamics and metabolite profiles using the same data presented in Fig 2. Implementation details of the CR model are provided in S1 Appendix. While the CR model offers the same physical constraints as the MiRNN, it lacks the flexibility of machine learning approaches since it assumes species dynamics are entirely governed by constant rates of competition and metabolite sharing of resources. Similar to the MiRNN, the LSTM offers greater flexiblity than the CR model due to the neural network architecture. The flexibility of neural network based models is governed by the number and dimension of hidden layers, which for the LSTM was chosen by Baranwal et al. to have a single hidden layer with 4096 nodes in order to predict both species abundances and metabolite concentrations. For all analyses in this study, the MiRNN and RNN use a single hidden layer with 16 units. Consequently, the LSTM proposed by Baranwal et al. when applied to a system with 25 species and 4 metabolites contains orders of magnitude more parameters (*n*_*θ*_ = 67, 735, 581) than the MiRNN and RNN (*n*_*θ*_ = 1, 245), and thus offers greater flexibility. However, the large number of model parameters in the LSTM model makes it challenging to perform the Bayesian parameter inference method used to train the MiRNN and RNN models [40], because this requires the computationally expensive task of inverting a matrix with dimension equal to the square of the number of parameters [12]. Therefore, alternative approaches for approximate Bayesian inference [40, 41] would need to be developed to apply the LSTM model for Bayesian optimization or experimental design. While the MiRNN did not outperform the LSTM on held out data from 20-fold cross-validation, the MiRNN displayed a higher median Pearson correlation for 22 of the 25 species (sign test, *p* < 1 × 10^−4^, *n* = 25) and for all four metabolites compared to the CR model (S3 Fig). To determine whether reducing the size of the training data favors the simpler CR model, we scanned a range of training sizes from 1 to 70 samples and found that all machine learning methods outperformed the CR model across this range (S4 Fig). In summary, the combined flexibility of the MiRNN and the incorporation of the physical constraint improved performance over both a mechanistic CR model and the unconstrained RNN model. Furthermore, reducing the number of parameters of machine learning approaches makes Bayesian inference methods tractable and thus enables a systematic method to determine model prediction uncertainty and optimize experimental designs.

We evaluated the quality of MiRNN prediction uncertainty on held-out data, since the ability to identify poorly understood conditions is crucial for selecting informative experimental designs that aim to fill knowledge gaps in the model. Evaluation of the log-likelihood of held-out testing data is a widely used approach to demonstrate a model’s ability to use prediction uncertainty to capture the variation in prediction error [42, 43]. Briefly, the log-likelihood (Eq 3) of held-out data will be higher when model predicted uncertainty is small for predictions that are close to measured values and when model prediction uncertainty is large for predictions that are further away from measured values (S5 Fig). We compared the log-likelihood of held-out data using a null model where the uncertainty in each prediction was estimated using a fixed variance, **Σ**_*y*_, to the log-likelihood using the condition-dependent model predicted variance given by Eq 9. The fixed estimate of **Σ**_*y*_ was computed using the expectation-maximization algorithm (S1 Appendix), which reflects the covariance in model prediction error on the training data. In this sense, **Σ**_*y*_ is the best guess of the variance that can be attributed to measurement noise. Uncertainty due to measurement noise cannot be reduced by collecting more data and is referred to as aleatory uncertainty, while uncertainty that could be minimized by collecting more data is referred to as epistemic uncertainty [44], both of which are captured by the model predicted uncertainty. The predicted uncertainty therefore reflects the degree of uncertainty associated with each experimental condition (e.g. the model could have varying levels of certainty about metabolite concentrations based on information from different consortia of microbial species). For the 10 randomized k-fold trials, the log-likelihood of held-out data using predicted variance was, on average, greater than the log-likelihood using the fixed variance (S5 Fig). The increase in the log-likelihood using model predicted uncertainty suggests that accounting for both aleatory and epistemic uncertainty improved estimates of the distribution of model predictions compared to a model that only accounted for aleatory uncertainty. The ability to assign greater uncertainty to less understood experimental conditions is a key attribute that enables efficient exploration of a high-dimensional experimental design space.

### 2.3 Optimization of the production of a key metabolite by a microbial community in a bioreactor

Mixed microbial communities cultured in bioreactors have many bioprocessing applications, including valorization of agricultural waste [45], production of medium chain fatty acids from carbon rich waste streams [1], and production of bioplastics as an alternative to petroleum-based plastics [46, 47]. Optimizing these functions requires manipulation of process control variables such as substrate feed rates, feed composition, pH, and gas exchange [48]. Although most bioprocessing applications have involved single organisms, microbial consortia have several advantages. These advantages include the ability to transform a wide range of available nutrients into valuable compounds by exploiting different metabolic niches and division of labor [49, 50] and robustness of target functions to environmental perturbations such as invasion [51–53].

**Fig 4:**
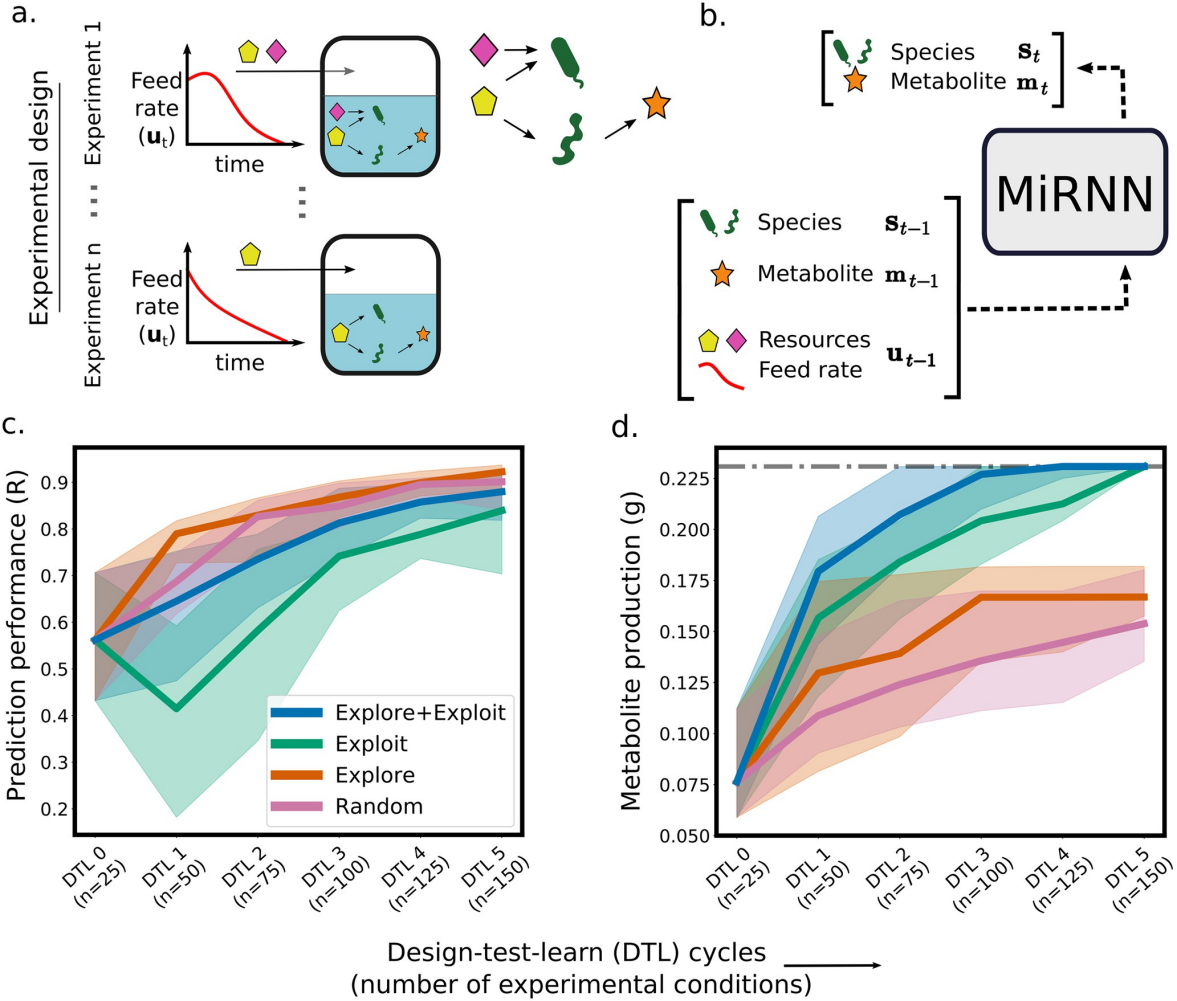
Optimization of resources and feed rate to maximize product. (a.) A schematic of the fed-batch bioreactor to be optimized, where the rate of a feed stream and the presence of resources in the feed (depicted as yellow and pink shapes) can both be adjusted in order to maximize production. Species (green shapes) that produce a valuable metabolite (orange star) compete for resources with species that do not produce the metabolite. (b.) A diagram that shows the inputs to the MiRNN model including species abundances, metabolite concentration, resource concentrations, and feed rate at time point *t* − 1. The model predicts species abundances and metabolite concentration at the next time step, *t*. Predicted species abundances and metabolite concentration are used as inputs to predict the next time step. (c.) A comparison of prediction performance (Pearson correlation coefficient) of end-point metabolite concentration between the proposed experimental design strategy that combines exploration and exploitation (blue) to pure exploitation (green), pure exploration (orange), and random sampling (purple). Solid lines show the median of the best recorded production (y-axis) up to each DTL cycle (x-axis) and uncertainty regions show the interquartile range computed over 30 trials each with random initial experimental designs. (d.) A comparison of metabolite maximization (showing median and interquartile range over 30 trials) between proposed exploration and exploitation (blue) to pure exploitation (green), pure exploration (orange), and random sampling (purple).

Resources (i.e. nutrients) are key control knobs for manipulating microbial community metabolism. Therefore, we consider selection of different combinations of resources and the rate at which the feed containing these resources is added to a fed-batch bioreactor containing a 5-member microbial community to maximize the production of a valuable metabolite (e.g. medium or long chain fatty acid [1]). Although our modeling framework is generally applicable to other reactor operation modes, such as continuous culture, we chose to study fed-batch operation to highlight the model’s ability to capture strong time-dependent changes in resources and biomass. Fed-batch operation involves a feed of substrates to the reactor without any discharge from the reactor. This in turn yields time-dependent variation in reactor volume, cell density, and product concentrations. This example demonstrates the ability of the MiRNN to optimize a multidimensional system with respect to control inputs that are both static (selection of resources) and dynamic (selection of the feed rate). As the ground truth system, we simulate a modified consumer-resource model [54] embedded in a fed-batch bioreactor model that assumes growth-associated kinetics for metabolite production (i.e. the rate of metabolite production is proportional to species growth rate) [55]. Species interactions are governed by competition for a limited set of resources. The governing equations of the ground-truth model are

$$\begin{array}{cc} \frac{dV}{dt} & =u\\ \frac{dr}{dt} & =r⊙(-s^{T}\cdot C-d)+\frac{u}{V}\left(r_{f}-r\right)\\ \frac{ds}{dt} & =s⊙(C\cdot r-g)-\frac{u}{V}\cdot s\\ \frac{dm}{dt} & =y_{m/s}^{T}\cdot \text{max}\left(0,\frac{ds}{dt}\right)-k_{d}\;m-\frac{u}{V}\;m \end{array}$$

where **r** is a vector of resource concentrations in the reactor, **r**_*f*_ is a vector of resource concentrations in the feed, **s** is a vector of species that utilize a subset of the resources, **d** is a vector of resource degradation rates, **g** is a vector of minimum growth rates needed for each species to survive, *m* is the metabolite concentration, **y**_*m*/*s*_ is a vector of yield coefficients, *k*_*d*_ is the metabolite degradation rate, [**C**]_*ij*_ is the rate species *i* consumes resource *j*, and *u*(*t*) represents the rate at which the feed is added to the bioreactor. Details on the specification of ground truth model parameters and the generation of simulated data are provided in the methods. Due to competition for limiting resources, introducing different combinations of resources will determine the temporal changes in species abundance. The objective for the MiRNN was to model species growth, **s**(*t*), and metabolite concentration, *m*(*t*), and identify the optimal combination of 7 resources, $r_{f}^{*}$, and the rate that the feed should be added over time, u*(*t*), in order to maximize the total amount of target metabolite, evaluated as the metabolite concentration multiplied by the reactor volume at the end of a 130 hour batch operation (Fig 4A and 4B). Simulated measurements were taken at 26 hour intervals, resulting in 5 measurements over the 130 hour simulation of the batch operation for each choice of resources. We considered 20 possible feed profiles for each possible combination of resources, which resulted in 20 × (2^7^ − 1) = 2, 540 possible resource and feed rate configurations and 5 × 2, 540 = 12, 700 experimental conditions including the 5 measurement times (S1 Appendix). This number of possible experimental conditions would not be feasible to exhaustively explore using generally low-throughput bioreactor systems. As such, it is necessary to develop methods to strategically navigate the experimental design space.

We compared the effectiveness of four different experimental design strategies (random sampling, pure exploration to maximize information content, pure exploitation to maximize profit, and exploration + exploitation to balance maximization of information content and profit) to find the bioreactor operating condition that maximized metabolite production. A pure exploration strategy seeks a set of experimental conditions that maximize the EIG, while the exploration + exploitation strategy evaluates experimental designs based on both the EIG and predicted outcomes. The variables subject to optimization included the resources in a feed stream and the rate at which the feed is added to a bioreactor over time (Fig 4A and 4B). Starting with a MiRNN trained on five randomly selected resource and feed rate configurations (25 experimental conditions, DTL 0), each experimental design method was used to select the next set of five resource and feed rate configurations that would compose the next experimental design (DTL 1). We emphasize that, in contrast to conventional Bayesian optimization approaches that select a single experimental condition in each cycle, the presented algorithm selects a batch of collectively informative experimental conditions in each experimental cycle. Data collected from each DTL cycle was used to update the model (Algorithm 1), which was then used to design the next DTL cycle, until five DTL cycles were completed resulting in a total of 30 resource and feed rate profiles tested (150 experimental conditions). This process was repeated 30 times each with a different randomly selected set of five resource and feed rate profiles in DTL 0.

After each round of training, model predictions of end-point metabolite concentrations were compared to ground truth values for all 2, 540 possible resource and feed rate profiles to gauge how well the model learns system behavior (Fig 4C and S6 Fig). A pure exploration strategy results in the most accurate model performance after training on data from the first experimental design, while a pure exploitation strategy results in a decrease in model prediction performance due to sampling of redundant experimental conditions in a narrow region of the design space. The production levels from each experimental design strategy shows that all model guided approaches (exploitation+exploration, exploitation, and exploration) navigate to higher metabolite producing operating conditions than a random sampling strategy. The model guided experimental design strategy that combines exploitation and exploration outperforms pure exploitation (Fig 4D), with significantly higher metabolite production in design cycles 1 (*p* = .0017), 2 (*p* = .0128), 3 (*p* = .0024), 4 (*p* = .0031), and 5 (*p* = .0203) according to a two-tailed paired t-test (n = 30). The median prediction performance of end-point metabolite concentration of the exploitation+exploration strategy after training on all data collected up to DTL 2 was not the highest across different strategies (*R* = 0.735). Nevertheless, the median identified metabolite production in the next design cycle (DTL 3) was nearly optimal (0.227 g). This implies that the model does not have to be highly accurate over the entire design space in order to be useful for seeking optimal operating conditions.

The ability of the MiRNN to predict both metabolite concentrations and species abundance over time can provide useful insight into the relationship between species abundances and system functions. This is in contrast to conventional Bayesian optimization approaches where a model (e.g. a Gaussian process) would be used to predict only metabolite production directly from the selection of resources and feed profile. To illustrate how predictions of species abundance can provide insights, we can analyze MiRNN predictions of species abundances and metabolite production for the condition that was predicted to maximize metabolite production. The MiRNN predicted both high metabolite production and relatively high growth of species *s*_2_ (S7 Fig), suggesting that species *s*_2_ produces the metabolite. This matches the ground truth model where the only species that produces the target metabolite is *s*_2_, ($y_{m/s}^{T}=\left[0,.5,0,0,0\right]$). This agreement between model predictions and the ground truth system suggests that, when ground truth is not known, analyzing MiRNN predictions of system behavior under different experimental conditions can provide meaningful insights.

## 3 Discussion

Despite their potential, the bottom-up design of microbiomes remains a challenge due to the massive design space of possible microbial consortia and environmental inputs (e.g. resources). Further, mechanisms driving community behaviors are typically not known precluding the development of predictive computational models based on first principles. In this work, we present the Microbiome Recurrent Neural Network (MiRNN); a physically constrained RNN model tailored to predict the dynamics of species interactions from data and predict target community functions. We use an approximate Bayesian inference strategy to compute a posterior parameter distribution, which enables the quantification of model prediction uncertainty and the evaluation of the information content of potential experimental designs. The ability of the MiRNN to learn microbial community dynamics from previously acquired data and evaluate the information content of experimental designs enables a sequential design-test-learn strategy to efficiently seek experimental conditions that optimize community functions (Fig 1).

Recent studies have underscored the need for an iterative (closed-loop) design-test-learn strategy to build computational models that enable efficient exploration and exploitation of biological systems [56, 57], and in particular, microbial communities [58, 59]. Toward this end, we introduce the first physically constrained machine learning model to predict the dynamics of microbial communities and show that incorporating a physical constraint significantly improved the model’s ability to predict species abundances and metabolite concentrations using experimental data. Further, the model yielded comparable prediction performance to a previously developed LSTM model [14] despite more than a 50,000 fold reduction in the number of model parameters (S3 Fig). When we analyzed the sensitivity of the LSTM and the MiRNN to the size of the training data, we found that prediction performance of species abundances continued to improve given more experimental data (S4 Fig), exemplifying the need for experimental design strategies to select the most informative data needed to train machine learning models. Toward this end, the reduction in model parameters of the MiRNN compared to the LSTM makes the use of Bayesian inference techniques more tractable for the purpose of quantifying model prediction uncertainty and evaluating the information content of experimental designs. Model prediction uncertainty is used in active learning [60, 61], Bayesian optimization [31], and reinforcement learning [48]. The framework differs from most previous work on optimal experimental design of biological systems [21, 22, 24] since it leverages model uncertainty to select a set of experimental conditions for the purpose of optimizing a function of interest (exploitation and exploration), as opposed to designing experiments for the sole purpose of refining a model (exploration). Our results demonstrate that while a pure exploration strategy is the best approach for improving model predictions, it does not efficiently seek conditions that optimize a system objective. However, the proposed experimental design strategy that combines exploration with exploitation reduces the number of experiments needed to find optimal conditions compared to exploitation alone (Fig 4C and 4D).

A limitation of the proposed methodology is that it relies on several approximations to enable fast selection of an experimental design, such as the assumption of Gaussian posterior parameter and predictive distributions. However, our model is an approximation of the ground truth system and despite imperfect predictions of system outcomes, the model is still able identify optimal experimental conditions (Fig 4). We therefore expect an approximate estimate of the information content of experimental designs to be sufficient in most applications. However, determining the effectiveness of our proposed experimental design framework to optimize systems where conditional distributions are known to be non-Gaussian [62] could be an area of future work. A limitation of the MiRNN, and any neural network based model, is that it offers limited interpretability for extracting new knowledge about the system. To tackle this problem, methods to extract meaning from a trained model such as Local Interpretable Model-agnostic Explanations (LIME) [63] have been used to derive relationships between variables in a similar modeling framework applied to microbial communities [14]. Alternatively, model predictions under different experimental conditions can be useful for gleaning mechanistic insights. For example, analyzing model predictions under the experimental condition that resulted in optimized metabolite production in our bioreactor case study correctly suggested that species *s*_2_ was responsible for producing the target metabolite (S7 Fig). Additionally, a limitation of discrete time models such as RNNs is the requirement for time series data to be sampled at consistent time intervals, which is often not the case in biological data sets where time series measurements are taken at different time resolutions. To overcome this limitation, the time interval can be included as an additional feature to the model or continuous time models such as neural ordinary differential equations [64] could be explored in future work.

Our framework enables the optimization of microbial community functions using a closed-loop Bayesian experimental design strategy. Our approach is capable of incorporating time dependent inputs (e.g. feed rate of a bioreactor) as potential controls to modulate system behavior. We note that although the constraint was incorporated for the purpose of modeling physically consistent bacterial growth, the same model could be applied to other chemical reaction networks that exhibit autocatalytic behaviors. For optimization of synthetic microbial communities, we envision future applications of this framework to include the selection of microbial species and resources (e.g. fibers) to accelerate the discovery of bacterial therapeutics that produce beneficial metabolites and display robustness to environmental perturbations. In addition, we could apply this framework to the design of microbial species and environmental conditions to increase biological nitrogen fixation for enhancing plant growth [65], and the design of microbial consortia with improved production of valuable chemicals such as medium-chain fatty acids and bioplastics from agricultural waste streams.

## 4 Methods

### 4.1 Microbiome Recurrent Neural Network (MiRNN) model

**Fig 5:**
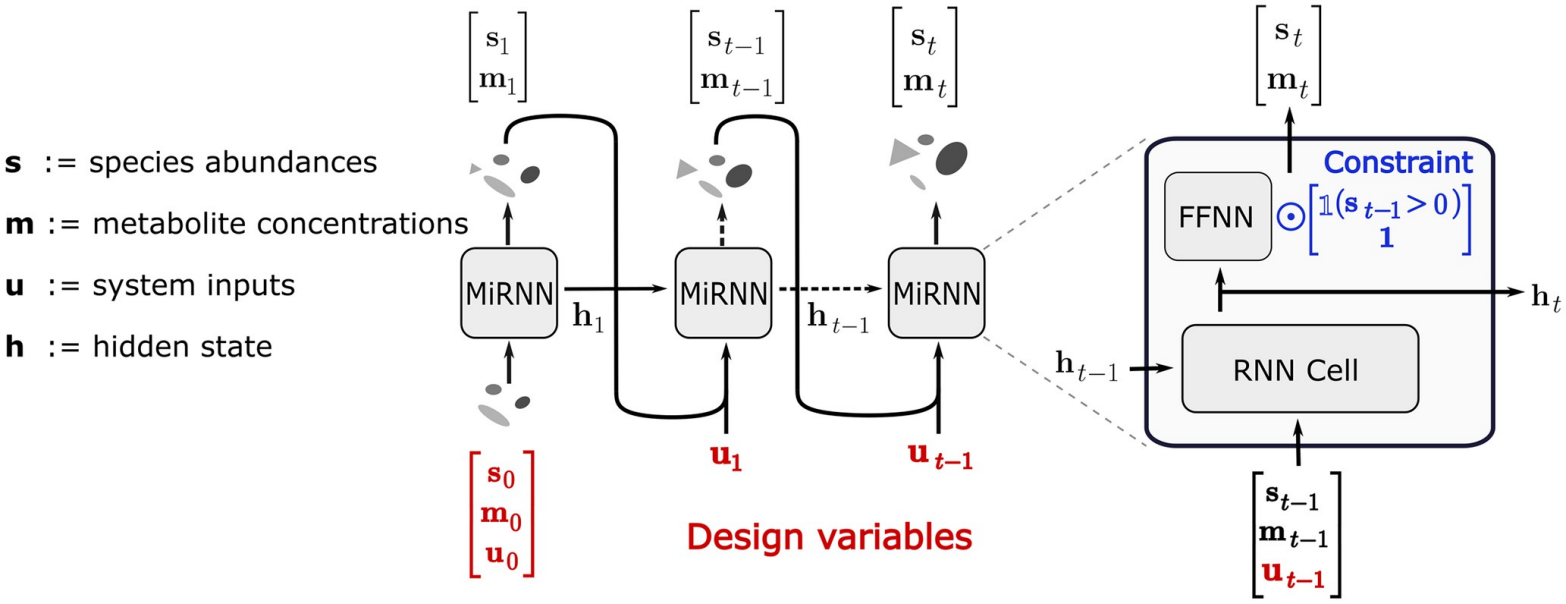
Microbiome Recurrent Neural Network architecture. Inputs to the RNN at time step *t* − 1 include the state of species abundances, metabolite concentrations, control inputs, and a latent vector that stores information from previous steps and whose dimension determines the flexibility of the model. The output from each MiRNN block is the predicted system state and the latent vector at the next time step, *t*. To avoid the physically unrealistic emergence of previously absent species, a constrained feed-forward neural network (FFNN) outputs zero valued species abundances if species abundances at the previous time step were zero.

RNNs are flexible machine learning models that can be applied to learn complex dynamical models directly from multivariate time series data. In this work, we present the Microbiome Recurrent Neural Network (MiRNN), illustrated in Fig 5, which is a modified RNN that aims to learn and predict the dynamic behavior of microbial communities. A more detailed description of the model architecture that shows activation functions and the architecture of the feed-forward neural network is shown in S1 Fig. Specifically, the MiRNN architecture aims to learn dynamic trajectories for species abundances and metabolites given a set of potentially time dependent inputs and encodes constraints that prevent prediction of negative species abundance or metabolite concentration and prevents the spontaneous emergence of a species.

We define a time horizon given by the index *t* = 0, …, *n*_*τ*_. The concentration of species at time *t* is denoted as $s_{t}\in R_{+}^{n_{s}}$. The concentration of metabolites at time *t* is denoted by $m_{t}\in R_{+}^{n_{m}}$. The value of the controls (inputs) at *t* is given by $u_{t}\in R^{n_{u}}$. The dynamic evolution of the MiRNN model is given by a mapping of the form:

$$\begin{array}{c} \left(s_{t},m_{t},h_{t}\right)=\text{MiRNN}(s_{t-1},m_{t-1},u_{t-1},u_{t},h_{t-1},\theta ),\;t=1,\ldots ,n_{\tau }. \end{array}$$ (1)

Here, $h_{t}\in R^{n_{h}}$ is a vector of latent variables at time *t* and $\theta \in R^{n_{\theta }}$ is a vector of model parameters. The latent variables propagate information from previous states in time. Increasing the dimension of the latent variable vector increases model complexity and flexibility, and can be selected using training data by maximizing the model evidence. For all analyses in this study, we set *n*_*h*_ = 16. We note that the controls at step *t* − 1 and *t* are both fed into the model evolution to account for the possibility of encountering strong time dependent variations in the control. The outputs of the RNN are the predicted system state and the latent vector at step *t*.

The set of model parameters of the architecture is composed of the weights and biases *θ* = {**W**_*hh*_, **b**_*hh*_, **W**_*ih*_, **W**_*ho*_, **b**_*ho*_, **h**_0_}, which are learned from data. The MiRNN architecture is designed to prevent the physically unrealistic emergence of previously absent microbial species; this is done by introducing a logic block that sets the species abundances to zero if the abundances at the previous time step were zero. Once the model is trained, a rectified linear unit activation function is applied to model outputs so that the state vector is strictly non-negative. Importantly, the data must be scaled to values between zero and one based on the maximum values in the training data so that zero abundance species remain zero. This is in contrast to standardizing features, which preprocesses input data by subtracting the mean and dividing by the standard deviation of each input [12], since this approach does not preserve zero-valued features. Preprocessing the data by dividing by the maximum value of each input enables the use of the constraint and allows the ReLU activation to eliminate the possibility of negative valued model outputs after applying the inverse scaling transformation.

In the context of experimental design, the control trajectories **u**_*t*_, *t* = 0, …, *n*_*τ*_ and the initial states **s**_0_ and **m**_0_ are variables that we can manipulate to influence the evolution of the state trajectories, **s**_*t*_ (species) and **m**_*t*_ (metabolites) for *t* = 1, …, *n*_*τ*_. Observed (measured) variables are referred to as outputs or observables; here, we assume that species abundances and metabolite concentrations can be measured and we encapsulate the entire set of output variables in the vector $y\in R^{n_{y}}$. The manipulated variables are called design variables in an experimental design context. We refer to a particular choice of design variables as an experimental condition, which is denoted by the variable *q*_*i*_. We define an experimental design as a set of *n* experimental conditions, **q** = {*q*_1_, …, *q*_*n*_}. We denote the entire set of *m* ≥ *n* possible experimental conditions as $Q=\{q_{1},\ldots ,q_{m}\}$.

### 4.2 Bayesian estimation and uncertainty quantification

We use a Bayesian framework to estimate the parameters of the model from designed experiments and to quantify the uncertainty of the model predictions given such experiments. We assume that we have an initial experimental design **q** with associated experimental conditions *q*_*i*_ indexed by *i* = 1, …, *n* each with corresponding observed outputs **y**(*q*_*i*_). The entire set of available data from an experimental design, **q**, is defined as the set $D\left(q\right)=\{y\left(q_{1}\right),\ldots ,y\left(q_{n}\right)\}$. We assume that the output observations are contaminated by random noise as:

$$\begin{array}{c} y\left(q_{i}\right)=M\left(\theta ,q_{i}\right)+\varepsilon ,\;i=1,\ldots ,n \end{array}$$ (2)

where $M(\theta ,q_{i})$ is the MiRNN output prediction at experimental condition *q*_*i*_, $\theta \in R^{n_{\theta }}$ are the MiRNN parameters, and *ε* is a noise random variable with probability density $p\left(\varepsilon \right)=N\left(0,\Sigma_{y}\right)$. The matrix **Σ**_*y*_ describes the variance of the noise (this is a hyper-parameter that can be defined manually or can be inferred from data (S1 Appendix)). We assume that the random noise over the multiple experiments is independent and identically distributed (i.i.d) and thus the model likelihood is given by:

$$\begin{array}{c} p\left(D\left(q\right)|\theta ,\Sigma_{y}\right)=\prod_{i=1}^{n}N\left(y\left(q_{i}\right)|M\left(\theta ,q_{i}\right),\Sigma_{y}\right). \end{array}$$ (3)

The prior over parameters is given by $p\left(\theta |\alpha \right)=N(0,\Sigma_{\theta }\left(\alpha \right))$, where $\Sigma_{\theta }\left(\alpha \right)\in R^{n_{\theta }\times n_{\theta }}$ is the prior covariance (a diagonal matrix) and $\alpha \in R_{+}^{n_{\theta }}$ is a tunable hyper-parameter vector that inflates/deflates this covariance (S1 Appendix). From Bayes’ theorem, the posterior parameter distribution is proportional to the product of the likelihood and the prior. The mode of the posterior density provides the *maximum a posteriori* (MAP) estimate of model parameters and is obtained by minimizing the negative log of the posterior density with respect to *θ*,

$$\begin{array}{c} \theta_{\text{MAP}}\left(q\right)=\underset{\theta }{\text{argmin}}\;\frac{1}{2}\sum_{i=1}^{n}{\left(y\left(q_{i}\right)-M\left(\theta ,q_{i}\right)\right)}^{T}\Sigma_{y}^{-1}\left(y\left(q_{i}\right)-M\left(\theta ,q_{i}\right)\right)+\frac{1}{2}\theta^{T}\Sigma_{\theta }{\left(\alpha \right)}^{-1}\theta , \end{array}$$ (4)

which we solve numerically using Newton’s method. The addition of the prior in the likelihood function encourages sparsity of the parameter estimates if *α* is made sufficiently large [12, 66].

In order to quantify the uncertainty of the parameter estimates, it is necessary to obtain their posterior density. Here, we use the so-called Laplace approximation; this assumes the posterior density is a Gaussian centered at *θ*_MAP_(**q**) and with covariance given by the inverse of the Hessian matrix of the negative log posterior, which is approximated as:

$$\begin{array}{c} H\left(q\right)=\Sigma_{\theta }{\left(\alpha \right)}^{-1}+\sum_{i=1}^{n}G{\left(q,q_{i}\right)}^{T}\Sigma_{y}^{-1}G\left(q,q_{i}\right), \end{array}$$ (5)

where $G\in R^{n_{y}\times n_{\theta }}$ is a matrix of derivatives of the model with respect to its parameters (referred to as the sensitivity matrix) and given by:

$$\begin{array}{c} G\left(q,q_{i}\right)≔\nabla_{\theta }\;M(\theta ,q_{i}){|}_{\theta =\theta_{\text{MAP}}\left(q\right)},\;i=1,\ldots ,n. \end{array}$$ (6)

Computation of gradients is performed using automatic differentiation with JAX [67] using Python 3. We note here that the Hessian matrix given by Eq 5 is full rank due to the inclusion of the diagonal prior precision matrix. The posterior predictive distribution of the outputs for any experimental condition q∈Q is found by marginalization over the posterior parameter distribution as:

$$\begin{array}{c} p\left(y\left(q\right)|D\left(q\right),\alpha ,\Sigma_{y}\right)=\int p\left(y\left(q\right)|\theta ,\Sigma_{y}\right)p\left(\theta |D\left(q\right),\alpha \right)d\theta . \end{array}$$ (7)

Obtaining an analytical expression for (7) requires linearization of the model prediction with respect to the parameters around *θ*_MAP_(**q**) to obtain a linear-Gaussian model [12],

$$\begin{array}{c} p\left(y\left(q\right)|D\left(q\right),\alpha ,\Sigma_{y}\right)\approx N\left(M\left(\theta_{\text{MAP}}\left(q\right),q\right),\Sigma_{y}\left(q\right)\right), \end{array}$$ (8)

with

$$\begin{array}{c} \Sigma_{y}\left(q\right)≔\Sigma_{y}+G\left(q,q\right)H{\left(q\right)}^{-1}G{\left(q,q\right)}^{T}. \end{array}$$ (9)

These expressions highlight how the design variables **q** propagate through the data D(q), the calculation of the estimate *θ*_MAP_(**q**), and ultimately influence the uncertainty of the model predictions. As such, it is important to derive systematic procedures to determine such experiments.

### 4.3 Fast Bayesian experimental design to optimize information content and system outcomes

Bayesian experimental design methods use information from a previous experimental design, **q**^(*l*)^, to inform the selection of the next experimental design, **q**^(*l*+1)^. One commonly used strategy is to find **q**^(*l*+1)^ that maximizes the expected information gain (EIG), which is quantified by the expected Kullback-Leibler divergence between the parameter posterior and the current parameter distribution [25, 61],

$$\begin{array}{c} \text{EIG}\left(q^{\left(l\right)},q^{\left(l+1\right)}\right)≔E_{D(q^{\left(l+1\right)})}[\text{KL}(p\left(\theta |D\left(q^{\left(l+1\right)}\right)\right)||p\left(\theta |D\left(q^{\left(l\right)}\right)\right)).] \end{array}$$ (10)

Using the model predictive distribution given by Eq 8 and assuming that the posterior distribution is Gaussian, the EIG can be approximated as (see S1 Appendix)

$$\begin{array}{cc} & \text{EIG}\left(q^{\left(l\right)},q^{\left(l+1\right)}\right)\approx \text{ln}\;\text{det}\;(H\left(q^{\left(l\right)}\right)+\sum_{i=1}^{n}G{\left(q^{\left(l\right)},q_{i}^{\left(l+1\right)}\right)}^{T}\Sigma_{y}^{-1}G\left(q^{\left(l\right)},q_{i}^{\left(l+1\right)}\right))-\text{ln}\;\text{det}\;(H(q^{\left(l\right)})). \end{array}$$ (11)

For linear-Gaussian models, experimental designs that maximize equation Eq 11 are referred to as Bayesian D-optimal [25], because they maximize the determinant of the expected posterior precision matrix. Similarly, D-optimal experimental designs are often selected based on the determinant of the Fisher information matrix (FIM) [68], given by

$$\begin{array}{c} \text{det}\;\text{FIM}\left(q^{\left(l\right)},q^{\left(l+1\right)}\right)≔\text{det}\;(\sum_{i=1}^{n}G{\left(q^{\left(l\right)},q_{i}^{\left(l+1\right)}\right)}^{T}\Sigma_{y}^{-1}G\left(q^{\left(l\right)},q_{i}^{\left(l+1\right)}\right)). \end{array}$$ (12)

Although widely used in practice [23, 69–71], methods for experimental design based on maximizing either Eqs 11 or 12 can be computationally expensive since they require evaluating the determinant of a matrix with dimensions *n*_*θ*_ × *n*_*θ*_. If the experimental design is composed of a single experimental condition, *q*^(*l*+1)^, it has been shown [25, 61] for linear-Gaussian models that the condition that maximizes the EIG is equivalent to the condition that maximizes the determinant of the prediction covariance due to the following identity (S1 Appendix),

$$\begin{array}{c} \text{ln}\;\text{det}\;(H\left(q^{\left(l\right)}\right)+G{\left(q^{\left(l\right)},q^{\left(l+1\right)}\right)}^{T}\Sigma_{y}^{-1}G\left(q^{\left(l\right)},q^{\left(l+1\right)}\right))-\text{ln}\;\text{det}\;(H(q^{\left(l\right)}))=\\ \text{ln}\;\text{det}\;(I_{n_{y}}+\Sigma_{y}^{-1}G\left(q^{\left(l\right)},q^{\left(l+1\right)}\right)H{\left(q^{\left(l\right)}\right)}^{-1}G{\left(q^{\left(l\right)},q^{\left(l+1\right)}\right)}^{T}). \end{array}$$ (13)

Since typically *n*_*y*_ << *n*_*θ*_, finding the experimental condition that maximizes prediction variance is a computationally efficient means of finding a Bayesian D-optimal condition; however, it is often desirable in experimental design applications to evaluate the information content of a set of *n* > 1 experimental conditions. We therefore present an expression that we show to be equivalent to Eq 11 (S1 Appendix) and which generalizes Eq 13 to compute the information content of *n* conditions,

$$\begin{array}{cc} \text{EIG}(q^{\left(l\right)},q^{\left(l+1\right)}) & \approx \text{ln}\;\text{det}\;(H\left(q^{\left(l\right)}\right)+\sum_{i=1}^{n}G{\left(q^{\left(l\right)},q_{i}^{\left(l+1\right)}\right)}^{T}\Sigma_{y}^{-1}G\left(q^{\left(l\right)},q_{i}^{\left(l+1\right)}\right))-\text{ln}\;\text{det}\;(H(q^{\left(l\right)}))\\ & =\sum_{i=1}^{n}\text{ln}\;\text{det}\;(I_{n_{y}}+\Sigma_{y}^{-1}G\left(q^{\left(l\right)},q_{i}^{\left(l+1\right)}\right)A_{i-1}^{-1}G{\left(q^{\left(l\right)},q_{i}^{\left(l+1\right)}\right)}^{T}) \end{array}$$ (14)

where

$$\begin{array}{c} A_{i}=A_{i-1}+G{\left(q^{\left(l\right)},q_{i}^{\left(l+1\right)}\right)}^{T}\Sigma_{y}^{-1}G\left(q^{\left(l\right)},q_{i}^{\left(l+1\right)}\right),\;A_{0}=H\left(q^{\left(l\right)}\right). \end{array}$$

The matrix inverse in Eq 14 can be efficiently computed using the Woodbury matrix identity,

$$\begin{array}{c} A_{i}^{-1}=A_{i-1}^{-1}-A_{i-1}^{-1}G{\left(q^{\left(l\right)},q_{i}^{\left(l+1\right)}\right)}^{T}{\left(\Sigma_{y}+G\left(q^{\left(l\right)},q_{i}^{\left(l+1\right)}\right)A_{i-1}^{-1}G{\left(q^{\left(l\right)},q_{i}^{\left(l+1\right)}\right)}^{T}\right)}^{-1}G\left(q^{\left(l\right)},q_{i}^{\left(l+1\right)}\right)A_{i-1}^{-1}. \end{array}$$ (15)

Using Eqs 14 and 15, we can efficiently approximate the EIG of an experimental design with *n* experimental conditions by avoiding computing the determinant of a matrix whose dimension is *n*_*θ*_ × *n*_*θ*_ in favor of evaluating the determinant and inverse of *n* matrices each with dimension *n*_*y*_ × *n*_*y*_ (S8 Fig).

In this work, we aim to find experiments that maximize information content and optimize a profit function of interest. As such, we define an acquisition function that accounts for the predicted profit of experimental outcomes [25] as well as the EIG,

$$\begin{array}{c} f\left(q^{\left(l\right)},q^{\left(l+1\right)}\right)=f_{P}\left(q^{\left(l\right)},q^{\left(l+1\right)}\right)+w_{I}\;\cdot \text{EIG}\left(q^{\left(l\right)},q^{\left(l+1\right)}\right) \end{array}$$ (16)

where *f*_*P*_(**q**^(*l*)^, **q**^(*l*+1)^) quantifies the predicted profit of the next design (e.g., total amount of product produced in each experiment). The profit function is an implicit function of the MiRNN model $M(\theta_{\text{MAP}}\left(q^{\left(l\right)}\right),q^{\left(l+1\right)})$ (i.e., the profit is predicted using the MiRNN model). The function EIG(**q**^(*l*)^, **q**^(*l*+1)^) quantifies the information content of the design **q**^(*l*+1)^) and is approximated using Eqs 14 and 15. The parameter $w_{I}\in R_{+}$ modifies the emphasis given to either profit or information content, and can be automatically adjusted to select for new experimental conditions as described in section 4.4. Given previously observed experimental designs **q**^(*l*)^ and a set Q of all possible experimental conditions that could be tested, our goal is to select the next design $q^{\left(l+1\right)}\subseteq Q$ such that we maximize the acquisition function:

$$\begin{array}{c} q^{*(l+1)}\in \underset{q^{\left(l+1\right)}\subseteq Q}{\text{argmax}}\;f\left(q^{\left(l\right)},q^{\left(l+1\right)}\right). \end{array}$$ (17)

We note that the optimal experiments q^*(*l*+1)^ are obtained based on best predicted performance (as predicted by the model); as such, these need to be tested in the real experimental system to obtain new outputs. This allows us to obtain a sequential experimental design framework in which we aim to progressively refine the model to maximize the profit function of interest.

**Algorithm 1**: Sequential Bayesian experimental design

**Require**: $D(q^{\left(0\right)})$, *f*_*P*_, *l*_max_

 *l* ← 0

 **while** *l* < *l*_max_ **do**

  {Estimate model parameter mean and covariance}

$\theta_{\text{MAP}}\left(q^{\left(l\right)}\right)\leftarrow {\text{argmax}}_{\theta }[-\text{ln}\;p(\theta |D\left(q^{\left(l\right)}\right))]$

$H\left(q^{\left(l\right)}\right)\leftarrow \Sigma_{\theta }^{-1}+\sum_{i}G{\left(q^{\left(l\right)},q_{i}^{\left(l\right)}\right)}^{T}\Sigma_{y}^{-1}G\left(q^{\left(l\right)},q_{i}^{\left(l\right)}\right)$

  {Design next experiment}

$q^{\left(l+1\right)}\leftarrow {\text{argmax}}_{q\subseteq Q}\;[\sum_{i}f_{P}\left(q^{\left(l\right)},q_{i}\right)+w_{I}\;\cdot \text{EIG}\left(q^{\left(l\right)},q\right)]$

  {Collect new data, append to existing data}

$D\left(q^{\left(l\right)}\right)\leftarrow \left\{D\left(q^{\left(l\right)}\right),D\left(q^{\left(l+1\right)}\right)\right\}$

  **q**^(*l*)^ ← {**q**^(*l*)^, **q**^(*l*+1)^}

  *l* ← *l* + 1

 **end while**

### 4.4 Greedy algorithm to search for optimal experimental designs

Finding the optimal next design q^*(*l*+1)^ requires an exhaustive search over the set Q (particularly when the design variables are categorical). As expected, however, exhaustive enumeration would require evaluating *f*(**q**^(*l*)^, **q**^(*l*+1)^) for all $q^{\left(l+1\right)}\subseteq Q$ (which can be computationally prohibitive). As such, we implement a greedy search algorithm that works satisfactorily well in practice. It is important to emphasize that the experimental design framework has the final goal of maximizing the profit function (as opposed to just refine the model); as such, it searches for experiments in a more targeted manner and can improve profit without having a perfect prediction model. Greedy algorithms are often used as an approximate approach to optimize experimental designs [22]. Given a total number of conditions to include in the next design, *n*_**q**^(*l*+1)^_, the search starts by finding an experimental condition that maximizes the profit function, $q_{1}^{\left(l+1\right)}\in \underset{q\in Q}{\text{argmax}}\;f_{p}\left(q^{\left(l\right)},q\right)$. With **q**^(*l*+1)^ initialized as $\left\{q_{1}^{\left(l+1\right)}\right\}$, the *i* > 1 experimental condition is selected by determining

$$\begin{array}{c} q_{i}^{\left(l+1\right)}=\underset{q\in Q}{\text{argmax}}\;f_{P}\left(q^{\left(l\right)},q\right)+w_{I}\cdot \text{EIG}\left(\left(q^{\left(l\right)},q^{\left(l+1\right)}\right),q\right) \end{array}$$ (18)

where *w*_*I*_ is set to a small initial value (e.g. .0001) and gradually increased until $q_{i}^{\left(l+1\right)}\notin q^{\left(l+1\right)}$. The process continues until a desired number of conditions are selected.

### 4.5 Specification of ground truth bioreactor model parameters

The governing equations of the model are given by

$$\begin{array}{cc} \frac{dV}{dt} & =u\\ \frac{dr}{dt} & =r⊙(-s^{T}\cdot C-d)+\frac{u}{V}\left(r_{f}-r\right)\\ \frac{ds}{dt} & =s⊙(C\cdot r-g)-\frac{u}{V}\cdot s\\ \frac{dm}{dt} & =y_{m/s}^{T}\cdot \text{max}\left(0,\frac{ds}{dt}\right)-k_{d}\;m-\frac{u}{V}\;m \end{array}$$

where **r** is a vector of resource concentrations in the reactor, **r**_*f*_ is a vector of resource concentrations in the feed, **s** is a vector of consumer species, **d** is a vector of resource degradation rates, **g** is a vector of minimum growth rates needed for each species to survive, *m* is the metabolite concentration, **y**_*m*/*s*_ is a vector of yield coefficients, *k*_*d*_ is the product degradation rate, [**C**]_*ij*_ is the rate species *i* consumes resource *j*, and *u*(*t*) represents the rate at which the feed is added to the reactor.

The parameters of the model that need to be specified include **C**, **d**, **g**, **y**_*m*/*s*_, and *k*_*d*_. The specification of the consumer resource component of the model and its parameters is a modified version of the model presented in [54]. The matrix of species-resource interaction coefficients was determined by first specifying the probability that a species depends on a resource, *p*_*s*/*r*_, which was set to .6 for the simulation. A matrix of *concentration parameters*, denoted as **Θ** determines the degree that a species depends on the concentration of a resource, where

$$\begin{array}{c} {\left[\Theta \right]}_{ij}={\left[\Theta^{\prime }\right]}_{ij}/\sum_{j=1}^{n_{r}}{\left[\Theta^{\prime }\right]}_{ij} \end{array}$$

where [**Θ**′]_*i*,*j*_ ∼ Uniform(0, 1) with probability *p*_*s*/*r*_ and is zero otherwise. The matrix of interaction coefficients are sampled from a Normal distribution with parameters given by

$$\begin{array}{c} {\left[C\right]}_{ij}∼N\left(\mu ={\left[\Theta \right]}_{ij},\sigma ={\left[\Theta \right]}_{ij}/10\right). \end{array}$$

All elements of the degradation rate of metabolites, **d**, and the minimum amount of resources necessary, **g**, were set to .01. The degradation rate of the product, *k*_*d*_, was set to .005. The metabolite yield coefficients were specified to be **y**_*m*/*s*_ = [0, .5, 0, 0, 0], since species two depended on the fewest number of resources. This specification is important so that the optimal set of resources in the feed is not simply the inclusion of all resources. Each output in each simulated condition was corrupted with 5% Gaussian noise to mimic variation in experimental measurements.

## Supporting information

S1 Fig Detailed view of MiRNN model architecture. The set of model parameters of the architecture is composed of the weights and biases *θ* = {**W**_*hh*_, **b**_*hh*_, **W**_*ih*_, **W**_*ho*_, **b**_*ho*_, **h**_0_}. The constraint uses an indicator function to determine whether the incoming species abundance vector, **s**_*t*−1_, is greater than zero. The effect of the constraint is to ensure that if a particular species is zero at time *t* − 1, the model prediction of that species at time *t* will also be zero. A LeakyReLU function is used to activate the hidden layer, and a ReLU output activation function ensures that model outputs are strictly non-negative once the model is trained. The ReLU is outlined by a dotted box to indicate that this activation is suppressed during training in order to penalize negative model predictions.
(TIF)

S2 Fig Examples of physically unrealistic species and metabolite predictions. (a.) Species and metabolite abundance cannot be negative. (b.) If a species is initially at zero abundance (i.e. not present), it cannot have a positive abundance at later time points.
(TIF)

S3 Fig Comparison of K-fold cross-validation performance. (a.) Comparison of LSTM (blue), CR (purple), RNN (green), and MiRNN (orange) prediction performance (coefficient of determination) of species abundances after performing 20-fold cross-validation over 10 trials, with the order of samples shuffled in each trial. Bar plot heights indicate the median prediction performance and error bars indicate the interquartile range computed over the 10 trials. (b.) Same as panel a, but comparing root-mean-squared-error (RMSE). (c.) Comparison of coefficient of determination of metabolite concentrations after performing 20-fold cross-validation over 10 trials, with the order of samples shuffled in each trial. Bar plot heights indicate the median prediction performance and error bars indicate the interquartile range computed over the 10 trials. (d.) Same as panel c, but comparing root-mean-squared-error (RMSE).
(TIF)

S4 Fig Sensitivity of prediction performance to training size. The median and interquartile range prediction performance of held-out samples over 10 random trials is plotted as the number of training samples increases over the range 1, 5, 10, 15, 20, 25, 30, 35, 40, 45, 50, 55, 60, 65, 70 samples. (a.) Average Pearson correlation (R) of species (b.) Average RMSE of species (c.) Average Pearson correlation of metabolites (d.) Average RMSE of metabolites.
(TIF)

S5 Fig Illustration of test data log-likelihood. Comparison of test data log-likelihood using predicted variance versus fixed variance. When deviations between measured and predicted values are high, a corresponding high prediction variance will improve the log-likelihood. Conversely, if deviations between measured and predicted values are small, then a small variance will improve the log-likelihood. (a.) The predicted variance captures variation between measured and predicted values resulting in a higher log-likelihood compared to panel (b.) where prediction uncertainty is based on a fixed estimate of the variance. (c.) Comparison of test data log-likelihood using predicted covariance (left) and fixed covariance (right) after performing 20-fold cross-validation over 10 trials. Bar plot heights indicate the median test data log-likelihood and error bars indicate the interquartile range computed over the 10 trials.
(TIF)

S6 Fig Comparison of experimental design strategies to improve root-mean-squared-error (RMSE). A comparison of prediction performance (RMSE) of end-point metabolite concentration between the proposed experimental design strategy that combines exploration and exploitation (blue) to pure exploitation (green), pure exploration (orange), and random sampling (purple). Solid lines show the median of the best recorded production (y-axis) up to each DTL cycle (x-axis) and uncertainty regions show the interquartile range computed over 30 trials each with random initial experimental designs.
(TIF)

S7 Fig Model predictions of the optimal experimental condition (Exp. 1). (a.) The heatmap shows which resources were included in each experimental condition, where dark blue indicates the presence of a resource in the feed stream. (b.) The set of feed rates in the experimental design. (c.) Observed metabolite production in the bioreactor for each experimental condition. (d.) Experimental condition one (Exp. 1) species predictions and uncertainty intervals (mean ±1 standard deviation) (e.) Experimental condition one (Exp. 1) metabolite prediction and uncertainty interval (mean ±1 standard deviation) (f.) Prediction (mean ±1 standard deviation) of metabolite production compared to measured values.
(TIF)

S8 Fig Evaluation times of different methods to compute the approximate information gain. Comparison of evaluation times of the expressions for the EIG, where the number of model parameters (*n*_*θ*_) is varied from 0 to 2500, *n*_*y*_ is the number of model outputs and *n* is the number of experimental conditions in the design.
(TIF)

S1 Appendix The appendix provides additional details on mathematical notation, data pre-processing, evaluation of model prediction performance, hyper-parameter optimization, the algorithm to estimate model parameters and hyper-parameters, justification of the experimental design information function, fast evaluation of the experimental design information function, implementation of the consumer resource model, and specification of the experimental design space for bioreactor optimization.
(PDF)
