# Supplementary figures and images for: Integrating a tailored recurrent neural network with Bayesian experimental design to optimize microbial community functions
Source: PLoS Comput Biol. 2023 Sep 29;19(9):e1011436. doi: 10.1371/journal.pcbi.1011436 (PMC10540976; doi:10.1371/journal.pcbi.1011436)

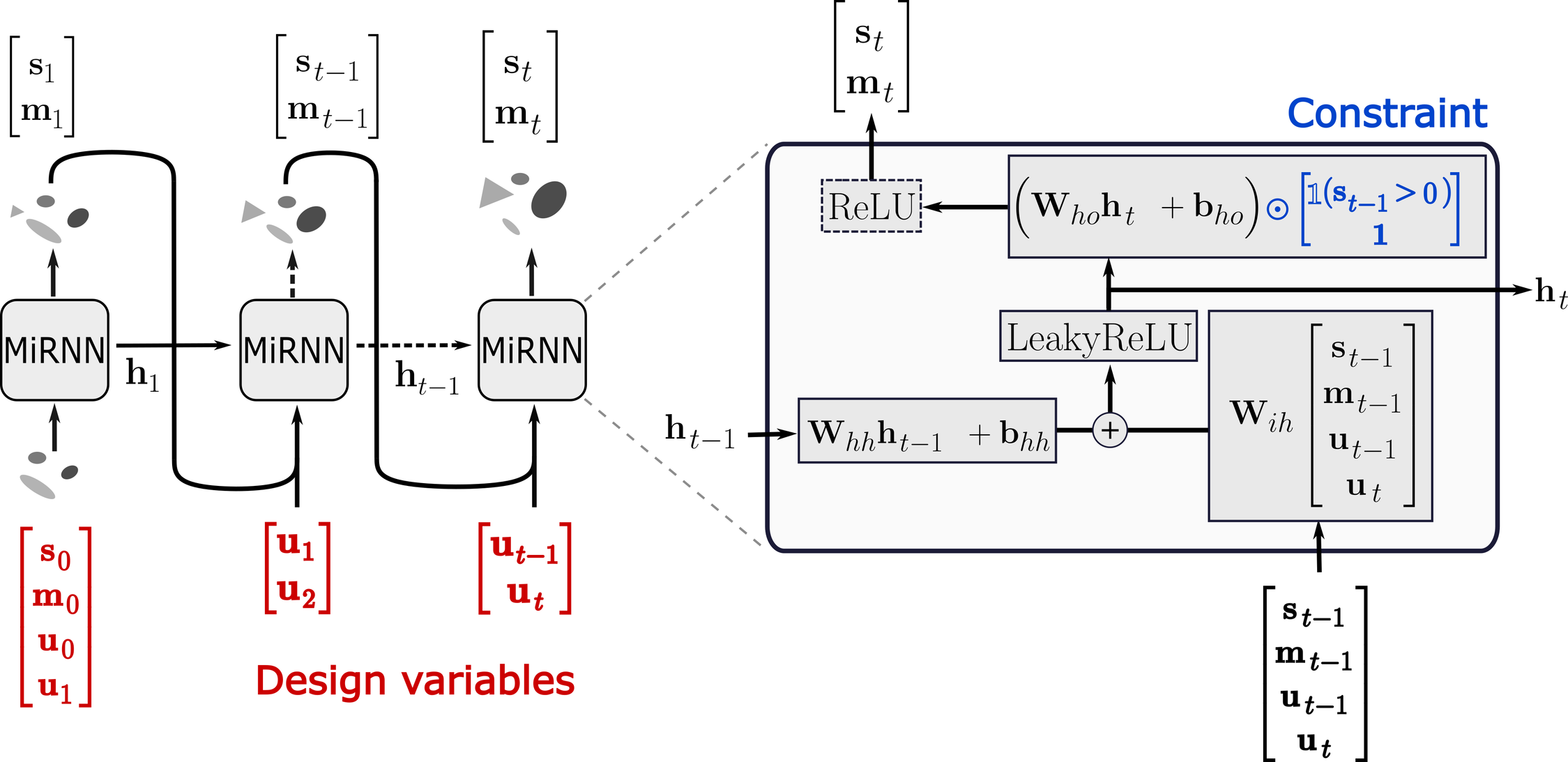

Supplement: S1 Fig — The set of model parameters of the architecture is composed of the weights and biases θ = {Whh, bhh, Wih, Who, bho, h0}. The constraint uses an indicator function to determine whether the incoming species abundance vector, st−1, is greater than zero. The effect of the constraint is to ensure that if a particular species is zero at time t − 1, the model prediction of that species at time t will also be zero. A LeakyReLU function is used to activate the hidden layer, and a ReLU output activation function ensures that model outputs are strictly non-negative once the model is trained. The ReLU is outlined by a dotted box to indicate that this activation is suppressed during training in order to penalize negative model predictions. (TIF) [file pcbi.1011436.s001.tif]

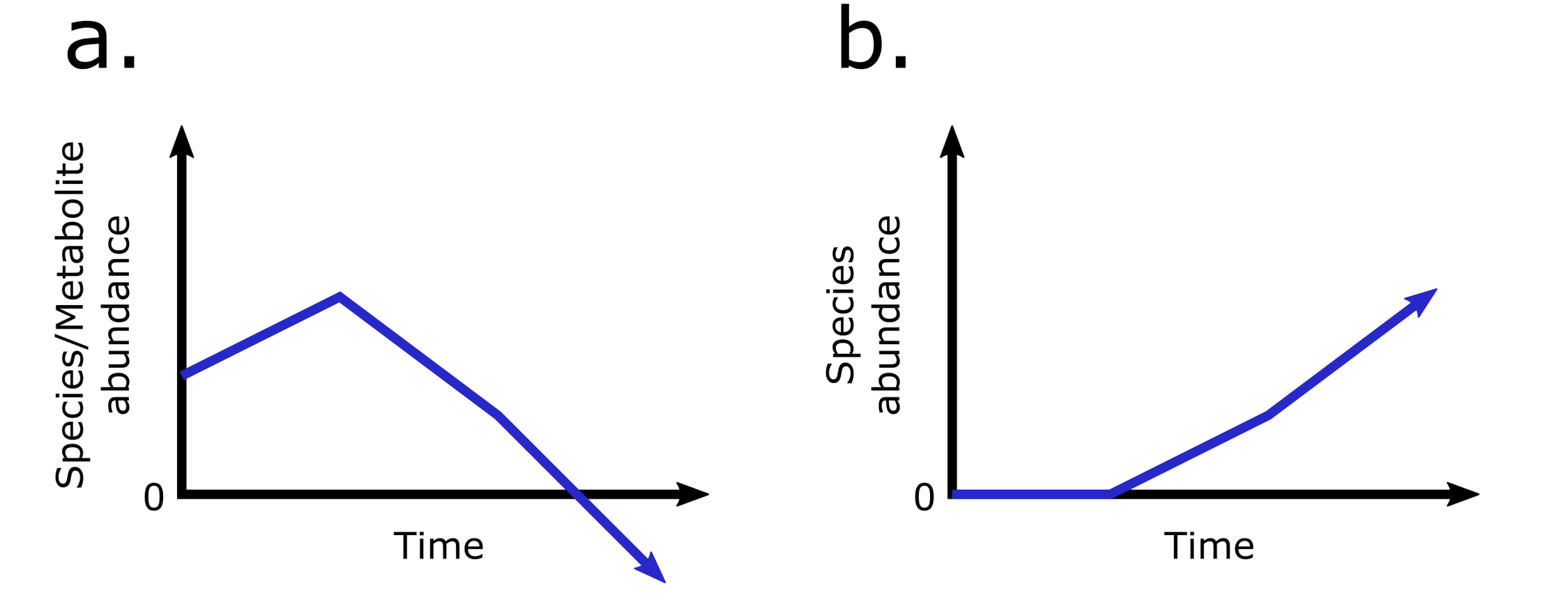

Supplement: S2 Fig — (a.) Species and metabolite abundance cannot be negative. (b.) If a species is initially at zero abundance (i.e. not present), it cannot have a positive abundance at later time points. (TIF) [file pcbi.1011436.s002.tif]

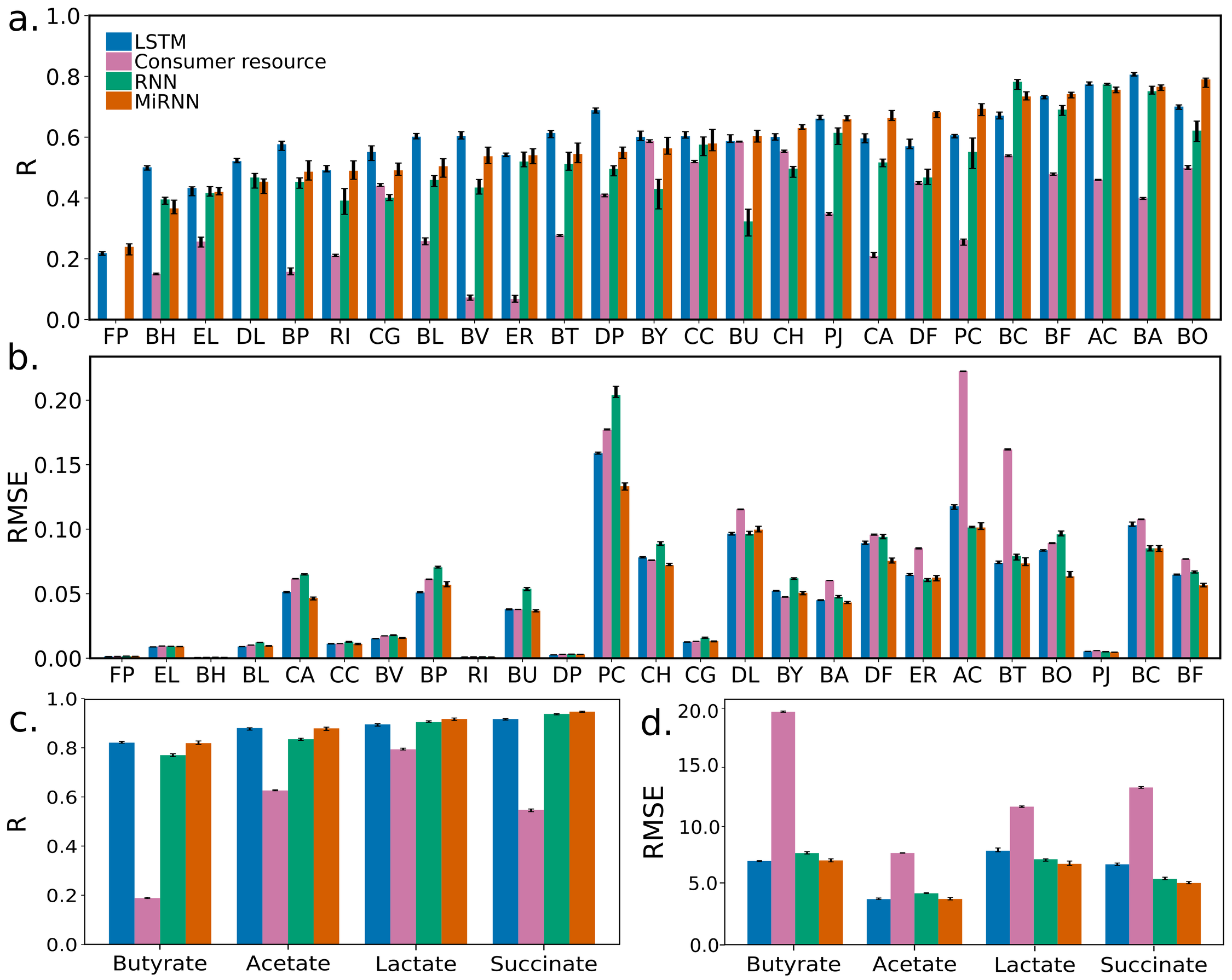

Supplement: S3 Fig — (a.) Comparison of LSTM (blue), CR (purple), RNN (green), and MiRNN (orange) prediction performance (coefficient of determination) of species abundances after performing 20-fold cross-validation over 10 trials, with the order of samples shuffled in each trial. Bar plot heights indicate the median prediction performance and error bars indicate the interquartile range computed over the 10 trials. (b.) Same as panel a, but comparing root-mean-squared-error (RMSE). (c.) Comparison of coefficient of determination of metabolite concentrations after performing 20-fold cross-validation over 10 trials, with the order of samples shuffled in each trial. Bar plot heights indicate the median prediction performance and error bars indicate the interquartile range computed over the 10 trials. (d.) Same as panel c, but comparing root-mean-squared-error (RMSE). (TIF) [file pcbi.1011436.s003.tif]

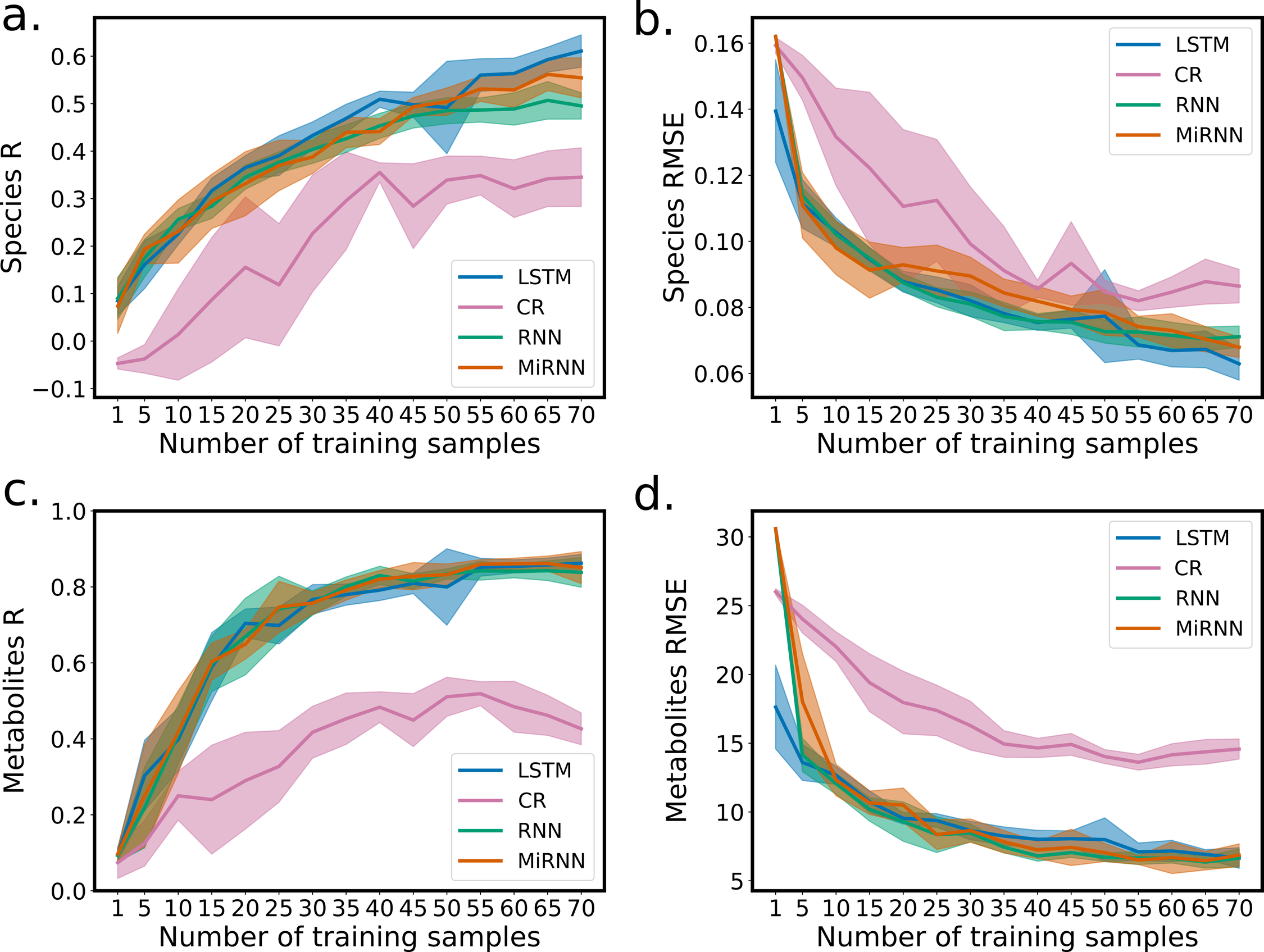

Supplement: S4 Fig — The median and interquartile range prediction performance of held-out samples over 10 random trials is plotted as the number of training samples increases over the range 1, 5, 10, 15, 20, 25, 30, 35, 40, 45, 50, 55, 60, 65, 70 samples. (a.) Average Pearson correlation (R) of species (b.) Average RMSE of species (c.) Average Pearson correlation of metabolites (d.) Average RMSE of metabolites. (TIF) [file pcbi.1011436.s004.tif]

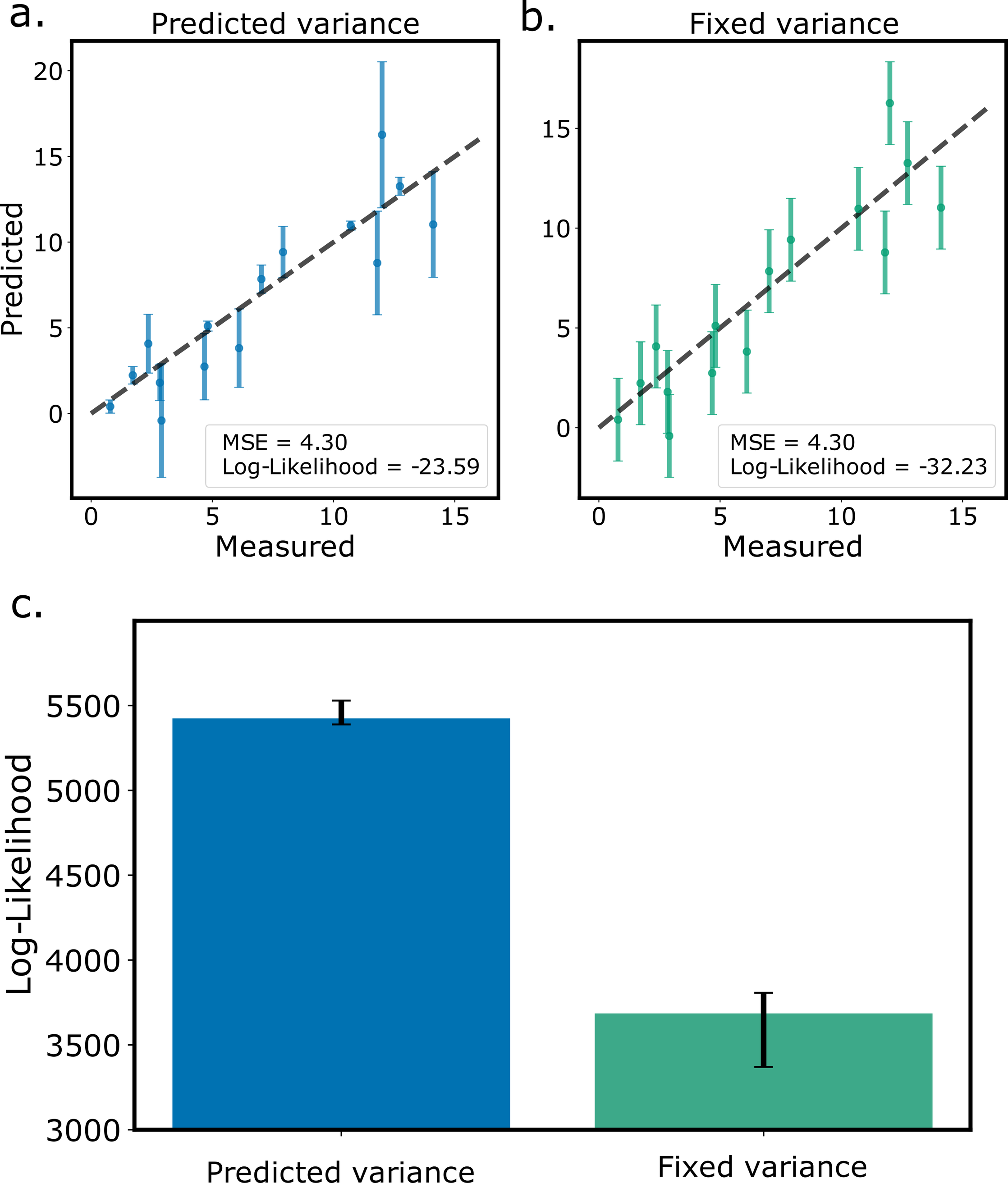

Supplement: S5 Fig — Comparison of test data log-likelihood using predicted variance versus fixed variance. When deviations between measured and predicted values are high, a corresponding high prediction variance will improve the log-likelihood. Conversely, if deviations between measured and predicted values are small, then a small variance will improve the log-likelihood. (a.) The predicted variance captures variation between measured and predicted values resulting in a higher log-likelihood compared to panel (b.) where prediction uncertainty is based on a fixed estimate of the variance. (c.) Comparison of test data log-likelihood using predicted covariance (left) and fixed covariance (right) after performing 20-fold cross-validation over 10 trials. Bar plot heights indicate the median test data log-likelihood and error bars indicate the interquartile range computed over the 10 trials. (TIF) [file pcbi.1011436.s005.tif]

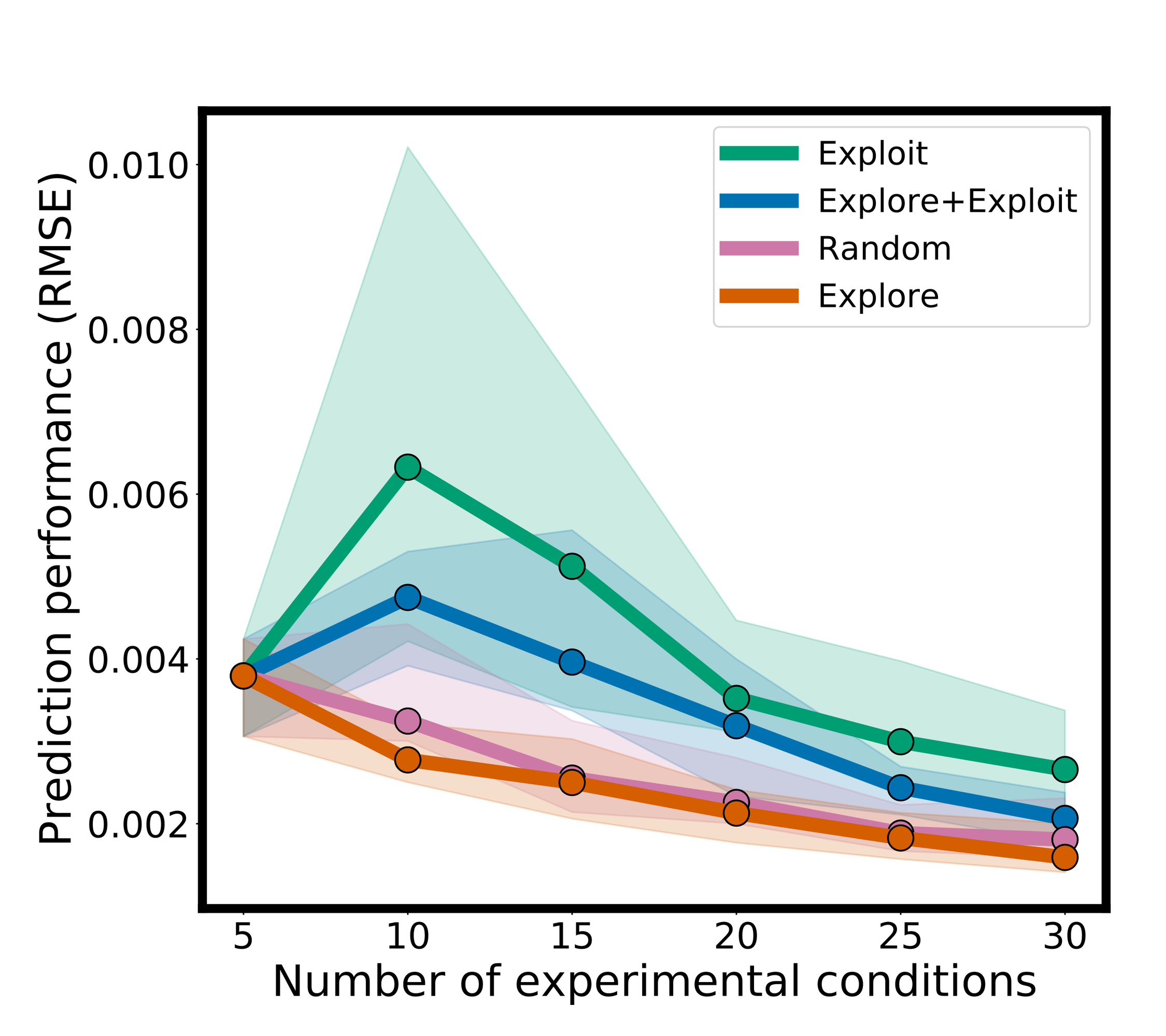

Supplement: S6 Fig — A comparison of prediction performance (RMSE) of end-point metabolite concentration between the proposed experimental design strategy that combines exploration and exploitation (blue) to pure exploitation (green), pure exploration (orange), and random sampling (purple). Solid lines show the median of the best recorded production (y-axis) up to each DTL cycle (x-axis) and uncertainty regions show the interquartile range computed over 30 trials each with random initial experimental designs. (TIF) [file pcbi.1011436.s006.tif]

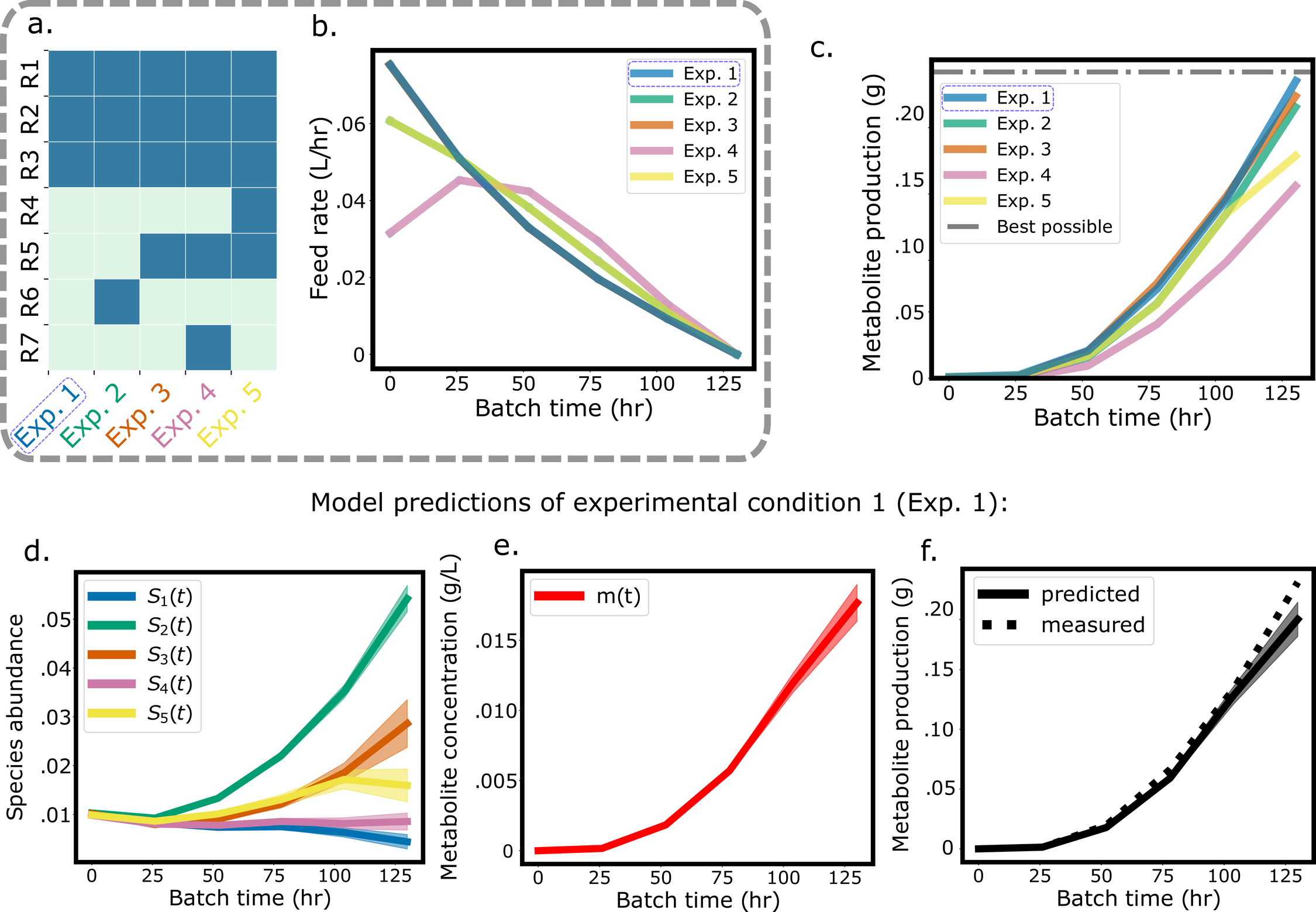

Supplement: S7 Fig — (a.) The heatmap shows which resources were included in each experimental condition, where dark blue indicates the presence of a resource in the feed stream. (b.) The set of feed rates in the experimental design. (c.) Observed metabolite production in the bioreactor for each experimental condition. (d.) Experimental condition one (Exp. 1) species predictions and uncertainty intervals (mean ±1 standard deviation) (e.) Experimental condition one (Exp. 1) metabolite prediction and uncertainty interval (mean ±1 standard deviation) (f.) Prediction (mean ±1 standard deviation) of metabolite production compared to measured values. (TIF) [file pcbi.1011436.s007.tif]

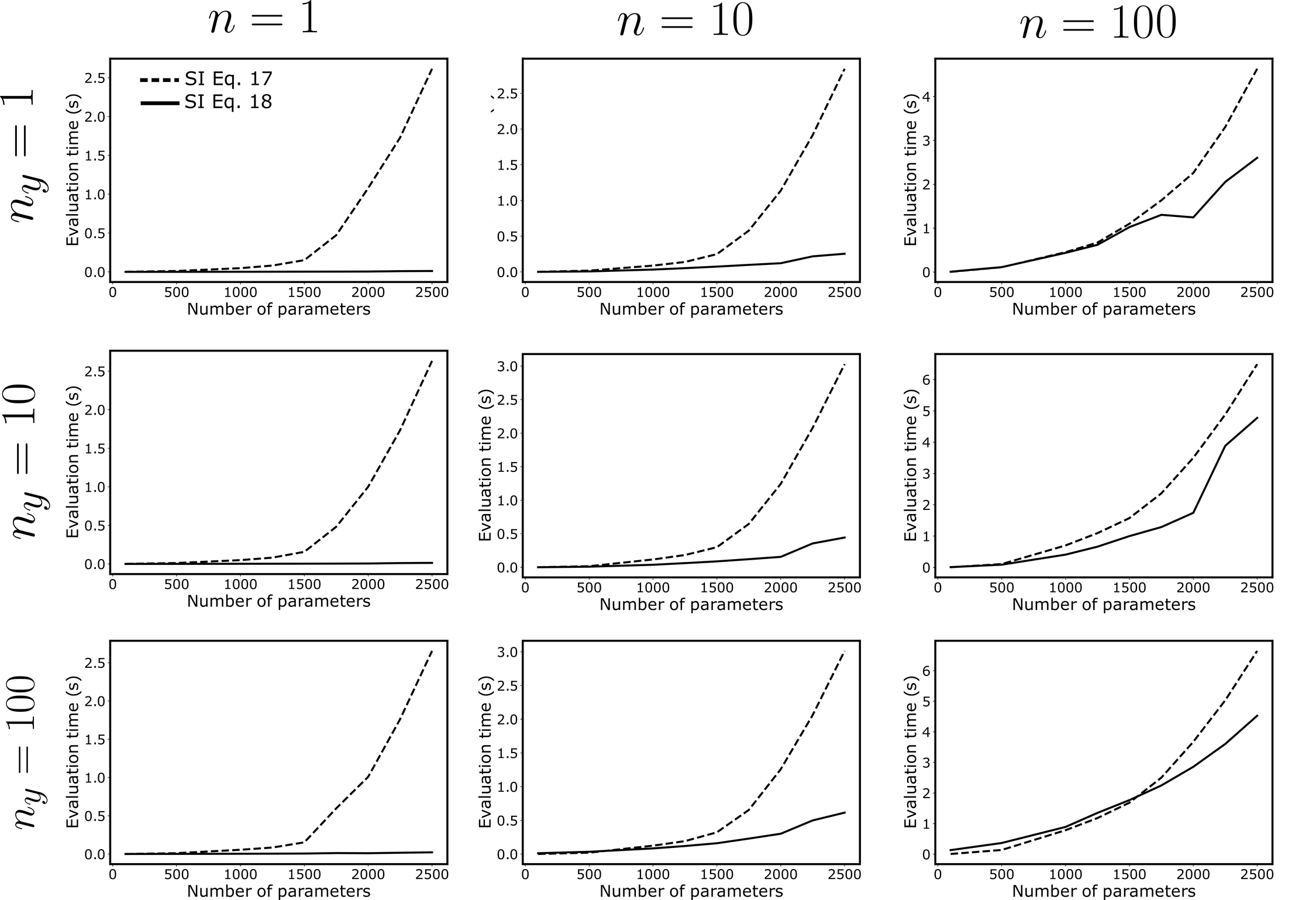

Supplement: S8 Fig — Comparison of evaluation times of the expressions for the EIG, where the number of model parameters (nθ) is varied from 0 to 2500, ny is the number of model outputs and n is the number of experimental conditions in the design. (TIF) [file pcbi.1011436.s008.tif]
