# Supplementary material for: Integrating a tailored recurrent neural network with Bayesian experimental design to optimize microbial community functions
Source: PLoS Comput Biol. 2023 Sep 29;19(9):e1011436. doi: 10.1371/journal.pcbi.1011436 (PMC10540976; doi:10.1371/journal.pcbi.1011436)
Supplement: S1 Appendix — The appendix provides additional details on mathematical notation, data pre-processing, evaluation of model prediction performance, hyper-parameter optimization, the algorithm to estimate model parameters and hyper-parameters, justification of the experimental design information function, fast evaluation of the experimental design information function, implementation of the consumer resource model, and specification of the experimental design space for bioreactor optimization. (PDF) [file pcbi.1011436.s009.pdf]

Supplementary Information for: Integrating a tailored recurrent neural network with Bayesian experimental design to optimize microbial community functions

Jaron C. Thompson[1,2], Victor M. Zavala[1], and Ophelia S. Venturelli[1,2,3*]

[1]Department of Chemical and Biological Engineering

[2]Department of Biochemistry

[3]Department of Bacteriology

University of Wisconsin-Madison, Madison, Wisconsin, 53706

[*]To whom correspondence should be addressed: venturelli@wisc.edu

1 SI Text

1.1 Nomenclature and Mathematical Notation

Symbol	Description
q	Experimental **condition**
	(e.g. A single inoculum and the time to measure species abundances after inoculation)
$\mathbf{q} = \{q_1, ..., q_n\}$	Experimental **design**
	(e.g. All inocula and measurement times in each well of a 96 well plate)
n	Number of unique conditions in an experimental design
$\mathcal{Q} = \{q_1, ..., q_m\}$	Set of all possible experimental conditions ($q \in \mathbf{q} \subseteq \mathcal{Q}$)
m	Number of possible experimental conditions
$f(\mathbf{q})$	Acquisition function that evaluates the utility of an experimental design
$\mathbf{s}_t$	Species abundances at time step t
$\mathbf{m}_t$	Metabolite abundances at time step t
$\mathbf{u}_t$	Controls (i.e. inputs) at time step t
$\mathbf{h}_t$	Latent variables at time step t
$\mathbf{y}(q)$	Measured species and metabolites that resulted from condition q
$\mathcal{D}(\mathbf{q})$	Set of measured outcomes from experimental design $\mathbf{q}$
$\mathcal{M}(\theta, q)$	Model predicted species and metabolites that resulted from condition q
θ	Model parameters
θ_{MAP}	Maximum a posteriori (MAP) estimate of model parameters
α	Diagonal entries of the prior parameter precision matrix
$\mathbf{\Sigma}_\theta(\alpha)$	Prior parameter covariance matrix
ε	zero-mean Gaussian random variable that models measurement noise
$\mathbf{\Sigma}_y$	Covariance matrix of Gaussian measurement noise
$\mathbf{\Sigma}_y(q)$	Covariance matrix of model predictive distribution of outcome due to condition q
$\mathbf{G}(\mathbf{q}, q)$	Gradient of a model trained on data from design $\mathbf{q}$ evaluated at condition q
$\mathbf{H}(\mathbf{q})$	Hessian of loss (negative log posterior) evaluated using data from design $\mathbf{q}$
$\text{EIG}(\mathbf{q}^{(l)}, \mathbf{q}^{(l+1)})$	Expected information gain of design $\mathbf{q}^{(l+1)}$ given data from design $\mathbf{q}^{(l)}$

1.2 Data pre-processing

To train the MiRNN, the data are pre-processed such that each species and each metabolite are first normalized by their maximum value in the training data. Given un-normalized training data, $\hat{\mathcal{D}}(\mathbf{q}) = \{\hat{\mathbf{y}}(q_1), ..., \hat{\mathbf{y}}(q_n)\}$, each output, indexed by $j = 1, ..., n_y$, is normalized so that

$$y_j(q_i) = \frac{\hat{y}_j(q_i)}{\max\{\hat{y}_j(q_k)\}_{k=1}^{n}} = \frac{\hat{y}_j(q_i)}{c_j} \tag{1}$$

where $\hat{y}_j(q_i)$ is the un-normalized (measured) output j from experimental condition q_i, $y_j(q_i)$ is the normalized output variable j from experimental condition q_i, and c_j is the maximum value of species j found in $\hat{\mathcal{D}}(\mathbf{q})$. When making predictions for any experimental condition q_i, the inverse transform is applied to the model output so that

$$\mathbb{E}[\hat{\mathbf{y}}(q_i)] = \text{diag}(\mathbf{c}) \cdot \mathcal{M}(\theta, q_i) \tag{2}$$

$$\text{Var}[\hat{\mathbf{y}}(q_i)] = \text{diag}(\mathbf{c}) \cdot \mathbf{\Sigma}_y(q_i) \cdot \text{diag}(\mathbf{c}) \tag{3}$$

1.3 Evaluation of model prediction performance

1.3.1 Pearson correlation coefficient

The Pearson correlation coefficient (R) was computed using the LINREGRESS function from SCIPY [11]. The Pearson correlation coefficient corresponding to prediction performance of output j over an un-normalized test data set, $\hat{\mathcal{D}}(\mathbf{q}) = \{\hat{\mathbf{y}}(q_1), ..., \hat{\mathbf{y}}(q_n)\}$ is given by

$$R_j = \frac{\sum_{i=1}^{n}(\hat{y}_j(q_i) - \frac{1}{n}\sum_{k=1}^{n}\hat{y}_j(q_k))(\mathbb{E}[\hat{y}_j(q_i)] - \frac{1}{n}\sum_{k=1}^{n}\mathbb{E}[\hat{y}_j(q_k)])}{\sqrt{\sum_{i=1}^{n}(\hat{y}_j(q_i) - \frac{1}{n}\sum_{k=1}^{n}\hat{y}_j(q_k))^2 \sum_{i=1}^{n}(\mathbb{E}[\hat{y}_j(q_i)] - \frac{1}{n}\sum_{k=1}^{n}\mathbb{E}[\hat{y}_j(q_k)])^2}} \tag{4}$$

1.3.2 Mean squared error

The mean square error between measured values and model predictions of output j is given by

$$MSE_j = \frac{1}{n}\sum_{i=1}^{n}(\hat{y}_j(q_i) - \mathbb{E}[\hat{y}_j(q_i)])^2. \tag{5}$$

1.3.3 Log-likelihood of test data

The log-likelihood is the log of the probability of a data set given the model. The log-likelihood evaluated for a data set, $\mathcal{D}(\mathbf{q})$, using model predicted covariance is given by

$$\begin{aligned}\ln p(\mathcal{D}(\mathbf{q})|\mathcal{M}(\theta,\mathbf{q}), \mathbf{\Sigma}_y(\mathbf{q})) = &-\sum_{i=1}^{n}(\mathbf{y}(q_i) - \mathcal{M}(\theta, q_i))^T \cdot \mathbf{\Sigma}_{\mathbf{y}}(q_i) \cdot (\mathbf{y}(q_i) - \mathcal{M}(\theta, q_i)) \\ &-\sum_{i=1}^{n}\frac{1}{2}\ln\det \mathbf{\Sigma}_{\mathbf{y}}(q_i) - \frac{n \cdot n_y}{2}\ln(2\pi)\end{aligned} \tag{6}$$

The log-likelihood evaluated using fixed covariance is given by

$$\begin{aligned}\ln p(\mathcal{D}(\mathbf{q})|\mathcal{M}(\theta,\mathbf{q}), \mathbf{\Sigma}_y) = &-\sum_{i=1}^{n}(\mathbf{y}(q_i) - \mathcal{M}(\theta, q_i))^T \cdot \mathbf{\Sigma}_{\mathbf{y}} \cdot (\mathbf{y}(q_i) - \mathcal{M}(\theta, q_i)) \\ &-\sum_{i=1}^{n}\frac{1}{2}\ln\det \mathbf{\Sigma}_{\mathbf{y}} - \frac{n \cdot n_y}{2}\ln(2\pi)\end{aligned} \tag{7}$$

1.4 Hyper-parameter optimization

We use an *empirical Bayes* [1] framework to infer model parameters and hyper-parameters where model parameters are optimized using Algorithm S1 and the parameter posterior density is approximated using the Laplace approximation [6]. To determine a set of optimal hyper-parameters, $\xi^* = \{\mathbf{\Sigma}_\theta^*, \mathbf{\Sigma}_y^*\}$, we seek ξ that maximizes the marginal likelihood function given by

$$p(\mathcal{D}(\mathbf{q})|\xi) = \int_\theta p(\mathcal{D}(\mathbf{q}), \theta|\xi) d\theta. \tag{8}$$

We can use the *expectation maximization* (EM) algorithm to update $\xi^{(l+1)}$, which involves maximizing the expected log likelihood,

$$\xi^{(l+1)} = \underset{\xi}{\text{argmax}}\ \mathbb{E}_{\theta|\mathcal{D}(\mathbf{q}),\xi^{(l)}}[\ln p(\mathcal{D}(\mathbf{q}), \theta|\xi)], \tag{9}$$

followed by re-evaluation of the posterior parameter distribution $p(\theta|\mathcal{D}(\mathbf{q}), \xi^{(l+1)})$. The process of maximizing Eq 9 and updating the posterior parameter distribution is repeated until convergence of the marginal likelihood function given by Eq 13.

Given an initial guess for the hyper-parameters, $\xi^{(l)}$, the posterior parameter distribution is approximated following the steps described in the methods section, *Bayesian estimation and uncertainty quantification*. With $\ln p(\mathcal{D}(\mathbf{q}), \theta) = \ln p(\mathcal{D}(\mathbf{q})|\theta) + \ln p(\theta)$, Eq 9 becomes

$$\mathbb{E}_{\theta|\mathcal{D}(\mathbf{q}),\xi^{(l)}}[\ln p(\mathcal{D}(\mathbf{q}), \theta|\xi)] = \mathbb{E}_{\theta|\mathcal{D}(\mathbf{q}),\xi^{(l)}}[\ln p(\mathcal{D}(\mathbf{q})|\theta, \xi)] + \mathbb{E}_{\theta|\mathcal{D}(\mathbf{q}),\xi^{(l)}}[\ln p(\theta|\xi)]. \tag{10}$$

The first term is the expectation of the log likelihood,

$$\begin{aligned}\mathbb{E}_{\theta|\mathcal{D}(\mathbf{q}),\xi^{(l)}}[\ln p(\mathcal{D}(\mathbf{q})|\theta, \xi)] = &-\frac{1}{2}\sum_{i=1}^{n} \mathbb{E}_{\theta|\mathcal{D}(\mathbf{q}),\xi^{(l)}}\left[(\mathbf{y}(q_i) - \mathcal{M}(\theta, q_i))^T \mathbf{\Sigma}_y^{-1}(\mathbf{y}(q_i) - \mathcal{M}(\theta, q_i))\right] \\ &- \frac{n}{2}\ln\left(2\pi \det \mathbf{\Sigma}_y\right).\end{aligned}$$

linearizing the model with respect to θ about θ_{MAP} and evaluating the expectation gives

$$\begin{aligned}\mathbb{E}_{\theta|\mathcal{D}(\mathbf{q}),\xi^{(l)}}[\ln p(\mathcal{D}(\mathbf{q})|\theta, \xi)] = &-\frac{1}{2}\sum_{i=1}^{n} \left(\mathbf{y}(q_i) - \mathcal{M}(\theta_{\text{MAP}}, q_i)\right)^T \mathbf{\Sigma}_y^{-1} \left(\mathbf{y}(q_i) - \mathcal{M}(\theta_{\text{MAP}}, q_i)\right) \\ &+ \text{Tr}\left(\mathbf{\Sigma}_y^{-1}\mathbf{G}(\mathbf{q}, q_i)\mathbf{H}(\mathbf{q})^{-1}\mathbf{G}(\mathbf{q}, q_i)^T\right) - \frac{n}{2}\ln\left(2\pi \det \mathbf{\Sigma}_y\right).\end{aligned}$$

Evaluating the second term in Eq 10 gives

$$\mathbb{E}_{\theta|\mathcal{D}(\mathbf{q}),\xi^{(l)}}[\ln p(\theta|\xi)] = -\frac{1}{2}\sum_{k=1}^{n_\theta}\left(\ln\,[\alpha]_k - [\alpha]_k \cdot ([\theta_{\text{MAP}}^2]_k + [\mathbf{H}(\mathbf{q})^{-1}]_{kk}) - \ln\,2\pi\right)$$

Update equations for ξ are found by taking the derivative of Eq 10 with respect to $\mathbf{\Sigma}_y$ and $[\alpha]_k$ and solving for $\mathbf{\Sigma}_y^{(l+1)}$ and $[\alpha^{(l+1)}]_k$,

$$\mathbf{\Sigma}_y^{(l+1)} = \frac{1}{n}\sum_{i=1}^{n}(\mathbf{y}(q_i) - \mathcal{M}(\theta_{\text{MAP}}, q_i))(\mathbf{y}(q_i) - \mathcal{M}(\theta_{\text{MAP}}, q_i))^T + \mathbf{G}(\mathbf{q}, q_i)\mathbf{H}(\mathbf{q})^{-1}\mathbf{G}(\mathbf{q}, q_i)^T, \tag{11}$$

$$[\alpha^{(l+1)}]_k = \frac{1}{\left([\theta_{\text{MAP}}^2]_k + [\mathbf{H}(\mathbf{q})^{-1}]_{kk}\right)}. \tag{12}$$

Model hyper-parameters are updated until convergence of the log of the model evidence (i.e. marginal likelihood), which is approximated using the Laplace approximation as

$$\begin{aligned}\ln p(\mathcal{D}(\mathbf{q})|\xi) \approx &-\frac{1}{2}\ln \det \mathbf{\Sigma}_\theta - \frac{n}{2}\ln 2\pi \det \mathbf{\Sigma}_y - \frac{1}{2}\ln \det \mathbf{H}(\mathbf{q}) - \frac{1}{2}\theta_{\text{MAP}}^T\mathbf{\Sigma}_y^{-1}\theta_{\text{MAP}} \\ &- \frac{1}{2}\sum_{i=1}^{n}(\mathbf{y}(q_i) - \mathcal{M}(\theta_{\text{MAP}}, q_i))^T\mathbf{\Sigma}_y^{-1}(\mathbf{y}(q_i) - \mathcal{M}(\theta_{\text{MAP}}, q_i)).\end{aligned} \tag{13}$$

1.5 Algorithm to estimate model parameters and hyper-parameters

Algorithm 1 provides pseudo-code for optimizing model parameters and hyper-parameters given an initial guess of the model parameters, θ, initial regularization parameter, α_0, learning rate l, tolerance on convergence of loss function, ϵ_1, tolerance on convergence of evidence, ϵ_2, and maximum number of times evidence convergence passes tolerance.

1.6 Function to quantify information content

Given a model that has been trained on previous data, $\mathcal{D}(\mathbf{q}^{(l)})$, where $\mathbf{q}^{(l)}$ represents all previous experimental designs, we wish to design the next experiment, $\mathbf{q}^{(l+1)}$. Using principles of Bayesian experimental design, the information content of the next experiment $\mathbf{q}^{(l+1)} \subset Q$ is evaluated using the expected gain in information that would result from updating the current model with a new dataset $\mathcal{D}(\mathbf{q}^{(l+1)}) = \{\mathbf{y}(q_1^{(l+1)}), ..., \mathbf{y}(q_n^{(l+1)})\}$ [10, 8]. The expected gain in information is the expected Kullback-Leibler divergence between the parameter posterior and current distribution, which is denoted as $p(\theta)$ in place of $p(\theta|\mathcal{D}(\mathbf{q}^{(l)}))$ to simplify the notation.

$$\begin{aligned}f_I(\mathbf{q}^{(l)}, \mathbf{q}^{(l+1)}) &:= \mathbb{E}_{\mathcal{D}(\mathbf{q}^{(l+1)})}[\text{KL}\left(p(\theta|\mathcal{D}(\mathbf{q}^{(l+1)}))||p(\theta)\right)] \\ &= \int_{\mathcal{D}(\mathbf{q}^{(l+1)})}\int_\theta p(\theta|\mathcal{D}(\mathbf{q}^{(l+1)}))\ln\left(\frac{p(\theta|\mathcal{D}(\mathbf{q}^{(l+1)}))}{p(\theta)}\right)\text{d}\theta\ p(\mathcal{D}(\mathbf{q}^{(l+1)}))\ \text{d}\mathcal{D}(\mathbf{q}^{(l+1)}) \\ &= \int_{\mathcal{D}(\mathbf{q}^{(l+1)})}\int_\theta \ln\left(p(\theta|\mathcal{D}(\mathbf{q}^{(l+1)}))\right)p(\theta, \mathcal{D}(\mathbf{q}^{(l+1)}))\text{d}\theta\ \text{d}\mathcal{D}(\mathbf{q}^{(l+1)}) - \int_\theta \ln\left(p(\theta)\right)p(\theta)\text{d}\theta \\ &= -h\left[\theta|\mathcal{D}(\mathbf{q}^{(l+1)})\right] + h\left[\theta\right]\end{aligned} \tag{14}$$

where $h[\theta]$ is the entropy of the current parameter distribution and $h\left[\theta|\mathcal{D}(\mathbf{q}^{(l+1)})\right]$ is the conditional entropy of the parameter distribution given the dataset $\mathcal{D}(\mathbf{q}^{(l+1)})$. Interpreting entropy as a measure

Require: θ, α_0, l, ϵ_1, ϵ_2, patience

loss $\leftarrow \infty$

evidence $\leftarrow -\infty$

passes $\leftarrow 0$

epoch $\leftarrow 0$

while passes $<$ patience **do**

{Update hyper-parameters}

if epoch = 0 **then**

$\mathbf{\Sigma}_\theta = 1/\alpha_0 \mathbb{I}_{n_\theta}$

$\mathbf{\Sigma}_y = \frac{1}{n}\sum_{i=1}^{n} \mathbf{y}(q_i)(\mathbf{y}(q_i))^T$

else

$\mathbf{\Sigma}_\theta = \text{diag}(\theta^2 + \text{diag}(\mathbf{H}(\mathbf{q})))$

$\mathbf{\Sigma}_y = \frac{1}{n}\sum_{i=1}^{n}(\mathbf{y}(q_i) - \mathcal{M}(\theta, q_i))(\mathbf{y}(q_i) - \mathcal{M}(\theta, q_i))^T + \mathbf{G}(\mathbf{q}, q_i)\mathbf{H}(\mathbf{q})^{-1}\mathbf{G}(\mathbf{q}, q_i)^T$

end if

{Compute MAP parameter estimate}

while MAP convergence $< \epsilon_1$ **do**

new loss $= \frac{1}{2}\sum_{i=1}^{n}(\mathbf{y}(q_i) - \mathcal{M}(\theta, q_i))^T \mathbf{\Sigma}_y^{-1}(\mathbf{y}(q_i) - \mathcal{M}(\theta, q_i)) + \frac{1}{2}\theta^T \mathbf{\Sigma}_\theta(\alpha)^{-1}\theta$

MAP convergence $=$ (loss $-$ new loss)$/\max(1, \text{loss})$

{Reduce learning rate if loss increases}

if MAP convergence < 0 **then**

$l = l/2$

end if

{Compute gradient, Hessian, descent direction}

$\mathbf{g} = \theta^T \mathbf{\Sigma}_\theta(\alpha)^{-1} + \sum_{i=1}^{n}(\mathbf{y}(q_i) - \mathcal{M}(\theta, q_i))^T \mathbf{G}(\mathbf{q}, q_i)$

$\mathbf{H}(\mathbf{q}) = \mathbf{\Sigma}_\theta(\alpha)^{-1} + \sum_{i=1}^{n} \mathbf{G}(\mathbf{q}, q_i)^T \mathbf{\Sigma}_y^{-1}\mathbf{G}(\mathbf{q}, q_i)$

$\mathbf{d} \leftarrow$ solve($\mathbf{H}(\mathbf{q}) \cdot \mathbf{d} = \mathbf{g}$)

{Update parameters, loss, epoch count}

$\theta = \theta - l \cdot \mathbf{d}$

loss = new loss

epoch = epoch + 1

end while

{Compute model evidence}

new evidence $= -\frac{1}{2}\ln \det \mathbf{\Sigma}_\theta - \frac{n}{2}\ln 2\pi \det \mathbf{\Sigma}_y - \frac{1}{2}\ln \det \mathbf{H}(\mathbf{q}) -$ loss

evidence convergence $=$ (evidence $-$ new evidence)$/\max(1, \text{abs(evidence)})$

if evidence convergence $< \epsilon_2$ **then**

evidence = new evidence

passes = passes + 1

$l = 2 * l$

end if

end while

Algorithm 1: Expectation maximization for inference of parameters and hyper-parameters

of uncertainty, the expected gain in information quantifies the amount the model expects the data, $\mathcal{D}(\mathbf{q}^{(l+1)})$, to reduce uncertainty in the model's current parameter values. Assuming that the posterior parameter distribution conditioned on $\mathcal{D}(\mathbf{q}^{(l+1)})$ is Gaussian with precision matrix, $\mathbf{H}(\mathbf{q}^{(l)}, \mathbf{q}^{(l+1)})$, we can analytically evaluate the integral over θ. Keeping only terms that depend on $\mathbf{q}^{(l)}$ and $\mathbf{q}^{(l+1)}$, this gives

$$
\begin{aligned}
-h[\theta|\mathcal{D}(\mathbf{q}^{(l+1)}))] &= \int_{\mathcal{D}(\mathbf{q}^{(l+1)})} \int_{\theta} \ln \left(p(\theta|\mathcal{D}(\mathbf{q}^{(l+1)})) \right) p(\theta|\mathcal{D}(\mathbf{q}^{(l+1)}))\mathrm{d}\theta \; p(\mathcal{D}(\mathbf{q}^{(l+1)})) \; \mathrm{d}\mathcal{D}(\mathbf{q}^{(l+1)}) \\
&= \int_{\mathcal{D}(\mathbf{q}^{(l+1)})} \ln \det \mathbf{H}(\mathbf{q}^{(l)}, \mathbf{q}^{(l+1)}) p(\mathcal{D}(\mathbf{q}^{(l+1)})) \; \mathrm{d}\mathcal{D}(\mathbf{q}^{(l+1)})
\end{aligned} \tag{15}
$$

We can once again make use of the Laplace approximation to get an expression for the posterior precision matrix, $\mathbf{H}(\mathbf{q}^{(l)}, \mathbf{q}^{(l+1)})$. Starting with Bayes' theorem, the parameter distribution conditioned on $\mathcal{D}(\mathbf{q}^{(l+1)})$ is proportional to the product of the likelihood of the data multiplied by the current parameter distribution

$$
p(\theta|\mathcal{D}(\mathbf{q}^{(l+1)})) \propto p(\mathcal{D}(\mathbf{q}^{(l+1)})|\theta)p(\theta),
$$

where $p(\theta) = \mathcal{N}(\theta_{\mathrm{MAP}}(\mathbf{q}), \mathbf{H}(\mathbf{q})^{-1})$. The Laplace approximation of the posterior parameter precision matrix is determined by taking the Hessian of the negative log of this posterior parameter distribution,

$$
\begin{aligned}
\mathbf{H}(\mathbf{q}^{(l)}, \mathbf{q}^{(l+1)}) &= -\nabla_\theta \nabla_\theta \ln p(\theta) - \nabla_\theta \nabla_\theta \ln \, p(\mathcal{D}(\mathbf{q}^{(l+1)})|\theta) \\
&= \mathbf{H}(\mathbf{q}^{(l)}) + \frac{1}{2} \nabla_\theta \nabla_\theta \sum_{i=1}^{n} (\mathbf{y}(q_i) - \mathcal{M}(\theta, q_i^{(l+1)}))^T \mathbf{\Sigma}_y^{-1} (\mathbf{y}(q_i) - \mathcal{M}(\theta, q_i^{(l+1)})) \\
&= \mathbf{H}(\mathbf{q}^{(l)}) + \sum_{i=1}^{n} \mathbf{G}(\mathbf{q}^{(l)}, q_i^{(l+1)})^T \mathbf{\Sigma}_y^{-1} \mathbf{G}(\mathbf{q}^{(l)}, q_i^{(l+1)}) \\
&\qquad + (\mathcal{M}(\theta, q_i^{(l+1)}) - \mathbf{y}(q_i))^T \mathbf{\Sigma}_y^{-1} \nabla_\theta \nabla_\theta \mathcal{M}(\theta, q_i^{(l+1)}).
\end{aligned}
$$

Because $\mathbf{y}(q_i)$ is modeled as a Gaussian with mean given by the model prediction, $\mathcal{M}(\theta, q_i^{(l+1)})$, for each $\mathbf{y}(q_i) \in \mathcal{D}(\mathbf{q}^{(l+1)})$, the summation over the residuals, $\mathcal{M}(\theta, q_i^{(l+1)}) - \mathbf{y}(q_i)$, vanishes when evaluating the expectation over $\mathcal{D}(\mathbf{q}^{(l+1)})$ in Eq. 15, assuming that the residuals are uncorrelated with the second derivative of the model with respect to parameters[1]. Evaluating Eq. 15 gives the final expression for the information function

$$
\mathrm{EIG}(\mathbf{q}^{(l)}, \mathbf{q}^{(l+1)}) \approx \ln \det \left(\mathbf{H}(\mathbf{q}^{(l)}) + \sum_{i=1}^{n} \mathbf{G}(\mathbf{q}^{(l)}, q_i^{(l+1)})^T \mathbf{\Sigma}_y^{-1} \mathbf{G}(\mathbf{q}^{(l)}, q_i^{(l+1)}) \right) - \ln \det \left(\mathbf{H}(\mathbf{q}^{(l)}) \right). \tag{16}
$$

Experimental designs that maximize Eq. 16 are called Bayesian D-optimal[9, 3].

1.7 Fast evaluation of the information function

Evaluation of the function given by Eq. 16 can be computationally expensive for models with a large number of parameters. Alternatively, we can compute the EIG using an equivalent expression,

$$\begin{aligned}\mathrm{EIG}(\mathbf{q}^{(l)}, \mathbf{q}^{(l+1)}) &\approx \ln\det\left(\mathbf{H}(\mathbf{q}^{(l)}) + \sum_{i=1}^{n} \mathbf{G}(\mathbf{q}^{(l)}, q_i^{(l+1)})^T \mathbf{\Sigma}_y^{-1} \mathbf{G}(\mathbf{q}^{(l)}, q_i^{(l+1)})\right) - \ln\det\left(\mathbf{H}(\mathbf{q}^{(l)})\right) \\ &= \sum_{i=1}^{n} \ln\det\left(\mathbb{I}_{n_y} + \mathbf{\Sigma}_y^{-1} \mathbf{G}(\mathbf{q}^{(l)}, q_i^{(l+1)}) \mathbf{A}_{i-1}^{-1} \mathbf{G}(\mathbf{q}^{(l)}, q_i^{(l+1)})^T\right) \end{aligned} \tag{17}$$

where

$$\mathbf{A}_i = \mathbf{A}_{i-1} + \mathbf{G}(\mathbf{q}^{(l)}, q_i^{(l+1)})^T \mathbf{\Sigma}_y^{-1} \mathbf{G}(\mathbf{q}^{(l)}, q_i^{(l+1)}), \qquad \mathbf{A}_0 = \mathbf{H}(\mathbf{q}^{(l)}). \tag{18}$$

Consequently, we can avoid taking the determinant of a matrix whose dimension is $n_\theta \times n_\theta$ in favor of evaluating the determinant and inverse of n matrices each with dimension $n_y \times n_y$ given $\mathbf{H}(\mathbf{q}^{(l)})^{-1}$. The matrix inverse $\mathbf{A}_i^{-1}$ can be evaluated efficiently using the Woodbury identity,

$$\mathbf{A}_i^{-1} = \mathbf{A}_{i-1}^{-1} - \mathbf{A}_{i-1}^{-1} \mathbf{G}(\mathbf{q}^{(l)}, q_i^{(l+1)})^T (\mathbf{\Sigma}_y + \mathbf{G}(\mathbf{q}^{(l)}, q_i^{(l+1)}) \mathbf{A}_{i-1}^{-1} \mathbf{G}(\mathbf{q}^{(l)}, q_i^{(l+1)})^T)^{-1} \mathbf{G}(\mathbf{q}^{(l)}, q_i^{(l+1)}) \mathbf{A}_{i-1}^{-1} \tag{19}$$

To see that the two expressions for the EIG in Eq. 17 are equivalent, we start with the following identity [1],

$$\det(\mathbb{I}_{n_\theta} + \mathbf{X}\mathbf{Z}^T) = \det(\mathbb{I}_{n_y} + \mathbf{X}^T \mathbf{Z}) \tag{20}$$

where $\mathbf{X}$ and $\mathbf{Z}$ have dimensions $n_\theta \times n_y$. Replacing terms with $\mathbf{X} = \mathbf{A}_{i-1}^{-1} \mathbf{G}(\mathbf{q}^{(l)}, q_i^{(l+1)})^T \mathbf{\Sigma}_y^{-1}$ and $\mathbf{Z}^T = \mathbf{G}(\mathbf{q}^{(l)}, q_i^{(l+1)})$, we get

$$\det(\mathbb{I}_{n_\theta} + \mathbf{A}_{i-1}^{-1} \mathbf{G}(\mathbf{q}^{(l)}, q_i^{(l+1)})^T \mathbf{\Sigma}_y^{-1} \mathbf{G}(\mathbf{q}^{(l)}, q_i^{(l+1)})) = \det(\mathbb{I}_{n_y} + \mathbf{\Sigma}_y^{-1} \mathbf{G}(\mathbf{q}^{(l)}, q_i^{(l+1)}) \mathbf{A}_{i-1}^{-1} \mathbf{G}(\mathbf{q}^{(l)}, q_i^{(l+1)})^T). \tag{21}$$

Using $\det(\mathbf{XZ}) = \det(\mathbf{X})\det(\mathbf{Z})$, and multiplying both sides of Eq. 21 by $\det(\mathbf{A}_{i-1})$, we have

$$\begin{aligned}&\det(\mathbf{A}_{i-1} + \mathbf{G}(\mathbf{q}^{(l)}, q_i^{(l+1)})^T \mathbf{\Sigma}_y^{-1} \mathbf{G}(\mathbf{q}^{(l)}, q_i^{(l+1)})) \\ &\qquad = \det(\mathbf{A}_{i-1}) \det(\mathbb{I}_{n_y} + \mathbf{\Sigma}_y^{-1} \mathbf{G}(\mathbf{q}^{(l)}, q_i^{(l+1)}) \mathbf{A}_{i-1}^{-1} \mathbf{G}(\mathbf{q}^{(l)}, q_i^{(l+1)})^T)\end{aligned} \tag{22}$$

Using $\mathbf{A}_i = \mathbf{A}_{i-1} + \mathbf{G}(\mathbf{q}^{(l)}, q_i^{(l+1)})^T \mathbf{\Sigma}_y^{-1} \mathbf{G}(\mathbf{q}^{(l)}, q_i^{(l+1)})$, we can express the summation in Eq. 17 as

$$\mathbf{H}(\mathbf{q}^{(l)}) + \sum_{i=1}^{n} \mathbf{G}(\mathbf{q}^{(l)}, q_i^{(l+1)})^T \mathbf{\Sigma}_y^{-1} \mathbf{G}(\mathbf{q}^{(l)}, q_i^{(l+1)}) = \mathbf{A}_{n-1} + \mathbf{G}(\mathbf{q}^{(l)}, q_n^{(l+1)})^T \mathbf{\Sigma}_y^{-1} \mathbf{G}(\mathbf{q}^{(l)}, q_n^{(l+1)}). \tag{23}$$

Using the identity in Eq. 22, we have

$$\det(\mathbf{A}_{n-1} + \mathbf{G}(\mathbf{q}^{(l)}, q_n^{(l+1)})^T \mathbf{\Sigma}_y^{-1} \mathbf{G}(\mathbf{q}^{(l)}, q_n^{(l+1)})) \\ = \det(\mathbf{A}_{n-1})\det(\mathbb{I}_{n_y} + \mathbf{\Sigma}_y^{-1} \mathbf{G}(\mathbf{q}^{(l)}, q_n^{(l+1)}) \mathbf{A}_{n-1}^{-1} \mathbf{G}(\mathbf{q}^{(l)}, q_n^{(l+1)})^T). \quad (24)$$

We can continue to apply Eq. 22 to $\mathbf{A}_{n-1} = \mathbf{A}_{n-2} + \mathbf{G}(\mathbf{q}^{(l)}, q_{n-1}^{(l+1)})^T \mathbf{\Sigma}_y^{-1} \mathbf{G}(\mathbf{q}^{(l)}, q_{n-1}^{(l+1)})$ until $n = 0$ and take the natural log of the result to yield Eq. 17.

1.8 Implementation of the consumer resource model

We used the consumer resource model as a benchmark for comparison with the MiRNN. The model structure is an adaptation of MacArthur's consumer resource model [4] that accounts for production and cross-feeding of metabolites,

$$\frac{d\mathbf{s}}{dt} = \mathbf{s} \odot \mathbf{f} \odot (\mathbf{C} \cdot \mathbf{m} - \mathbf{g}) \\ \frac{d\mathbf{m}}{dt} = \mathbf{P} \cdot \mathbf{s} - \mathbf{m} \odot \left(\mathbf{s}^T \cdot \mathbf{C}\right) \quad (25)$$

where $\mathbf{s} \in \mathbb{R}_+^{n_s}$ is a vector of species abundances, $\mathbf{m} \in \mathbb{R}_+^{n_m}$ is a vector of metabolite concentrations, $[\mathbf{C}]_{ij} \in \mathbb{R}_+$ is the rate that species i consumes resource j, $g_i \in \mathbb{R}_+$ is the minimum amount of resource consumption required by species i to maintain a positive growth rate, $f_i \in [0, 1]$ is the efficiency of species i to convert a resource excess into growth, and $[\mathbf{P}]_{ij} \in \mathbb{R}_+$ is the production of metabolite i by species j. To constrain species efficiencies to be between zero and one, a sigmoid function is used, where $\mathbf{f} = \sigma(\hat{\mathbf{f}})$. Because all other model parameters are strictly non-negative values, unconstrained parameter optimization was performed on log transformed values, $\theta = \{\hat{\mathbf{f}}, \log_2(\mathbf{g}), \log_2(\mathbf{C}), \log_2(\mathbf{P})\}$. Numerical integration was performed using the ODEINT function provided by JAX[2] and inference of parameters and hyper-parameters was performed using Algorithm S1. Prior to parameter estimation, metabolites were scaled by dividing by the largest measured metabolite value in the training data. In addition to the four measured metabolites (acetate, butyrate, lactacte, and succinate), a fifth metabolite with an initial value of one was included to account for the presence of additional carbon sources such as glucose, maltose, and other sugars in the media [5].

1.9 Specification of experimental design space for bioreactor optimization

We build on the dynamic design of experiments approach outlined in [7] to propose candidate feed-flowrate profiles as a linear combination of orthogonal basis functions. We note that while this is a useful approach for generating feasible control profiles, it is not a requirement for the presented method since the MiRNN is directly a function of the control profile and not the parameters that encode the profile. The batch time for the reactor was set to 130 hours, the initial reactor volume is set to 7L, and a limit on the reactor volume is set to 10L. The limit on reactor volume imposes a constraint on the possible feed-flowrate profiles where

$$V(0) + \int_0^{t_{\text{batch}}} u(t)\text{d}t \leq V_{\text{max}}. \quad (26)$$

By defining a dimensionless time, $\tau = t/t_{\text{batch}}$, and following the steps outlined in [7], we use the following equation to encode different time dependent feed-flowrates,

$$u(\tau) = \frac{6}{130}(1-\tau)(1 + x_1 P_0(\tau) + x_2 P_1(\tau) - (x_1 + x_2)P_2(\tau)), \tag{27}$$

where $P_i(\tau)$ is the ith Legendre polynomial and $-.5 \leq x_1 \pm x_2 \leq .5$. Selecting a different set of the coefficients, x_i, results in different feasible feed-flowrate profiles. A set of 20 different feed flow rates was generated using latin-hypercube sampling of each x_i. The design space, Q, was composed of matching each of the 20 feed flow rates with every possible combination of resources (excluding no resources), which resulted in a set of $20 \times (2^7 - 1) = 2,540$ experimental conditions. Each output in each simulated condition was corrupted with 5% Gaussian noise to mimic variation in experimental measurements.

References

[1] Christopher M Bishop and Nasser M Nasrabadi. *Pattern recognition and machine learning*. Springer, 2006.

[2] James Bradbury, Roy Frostig, Peter Hawkins, Matthew James Johnson, Chris Leary, Dougal Maclaurin, George Necula, Adam Paszke, Jake VanderPlas, Skye Wanderman-Milne, and Qiao Zhang. JAX: composable transformations of Python+NumPy programs, 2018.

[3] Kathryn Chaloner and Isabella Verdinelli. Bayesian experimental design: A review. *Statistical Science*, 10:273–304, 1995.

[4] Peter Chesson. Macarthur's consumer-resource model. *Theoretical Population Biology*, 37(1):26–38, 1990.

[5] Ryan L Clark, Bryce M Connors, David M Stevenson, Susan E Hromada, Joshua J Hamilton, Daniel Amador-Noguez, and Ophelia S Venturelli. Design of synthetic human gut microbiome assembly and butyrate production. *Nature communications*, 12(1):1–16, 2021.

[6] F Dan Foresee and Martin T Hagan. Gauss-newton approximation to bayesian learning. In *Proceedings of international conference on neural networks (ICNN'97)*, volume 3, pages 1930–1935. IEEE, 1997.

[7] Christos Georgakis. Design of dynamic experiments: A data-driven methodology for the optimization of time-varying processes. *Industrial & Engineering Chemistry Research*, 52(35):12369–12382, 2013.

[8] Juliane Liepe, Sarah Filippi, Michał Komorowski, and Michael P. H. Stumpf. Maximizing the information content of experiments in systems biology. *PLOS Computational Biology*, 9(1):1–13, 01 2013.

[9] Ali Shahmohammadi and Kimberley B McAuley. Using prior parameter knowledge in model-based design of experiments for pharmaceutical production. *AIChE Journal*, 66(11):e17021, 2020.

[10] Isabella Verdinelli and Joseph B Kadane. Bayesian designs for maximizing information and outcome. *Journal of the American Statistical Association*, 87(418):510–515, 1992.

[11] Pauli Virtanen, Ralf Gommers, Travis E. Oliphant, Matt Haberland, Tyler Reddy, David Cournapeau, Evgeni Burovski, Pearu Peterson, Warren Weckesser, Jonathan Bright, Stéfan J. van der Walt, Matthew Brett, Joshua Wilson, K. Jarrod Millman, Nikolay Mayorov, Andrew R. J. Nelson, Eric Jones, Robert Kern, Eric Larson, C J Carey, İlhan Polat, Yu Feng, Eric W. Moore, Jake VanderPlas, Denis Laxalde, Josef Perktold, Robert Cimrman, Ian Henriksen, E. A. Quintero, Charles R. Harris, Anne M. Archibald, Antônio H. Ribeiro, Fabian Pedregosa, Paul van Mulbregt, and SciPy 1.0 Contributors. SciPy 1.0: Fundamental Algorithms for Scientific Computing in Python. *Nature Methods*, 17:261–272, 2020.
